# Self‐Report Questionnaires to Measure Big Five Personality Traits in Children and Adolescents: A Systematic Review

**DOI:** 10.1111/sjop.13110

**Published:** 2025-04-01

**Authors:** Giada Vicentini, Daniela Raccanello, Roberto Burro

**Affiliations:** ^1^ Department of Human Sciences University of Verona Verona Italy

**Keywords:** adolescents, Big Five, children, self‐report, systematic review

## Abstract

Personality can be described by referring to the so‐called Big Five traits, that is, Extraversion, Agreeableness, Conscientiousness, Neuroticism, and Openness. These dimensions contribute to explaining individual differences not only in adults but also in children and adolescents. Although many authors used adult or other‐report instruments to assess the Big Five in young people, others developed or adapted specific self‐report measures for them. A systematic overview of research articles developing or validating self‐report questionnaires to measure the Big Five traits in children and/or adolescents is currently absent. Accordingly, a review of the literature seems necessary to better guide practitioners and researchers interested in this assessment. We consulted PsycINFO, PubMed, and Scopus. We extracted 20 articles from the initial 1449 records and another 4 articles by consulting their references. They described 10 questionnaires developed for 7–18‐year‐olds (with one exception). We provided detailed summaries of their language, trait labels, facets, length, item types, response scale, and item development or selection procedure. The structural validity and internal consistency of the selected questionnaires were evaluated by adapting the COSMIN guideline. All the questionnaires reflect an attempt to consider the cognitive skills and individual experiences that characterize children and/or adolescents. However, our findings highlighted some limitations in the validity or reliability of some of them. These results can provide guidance for selecting the most appropriate instrument depending on the contextual needs and for developing or adapting new questionnaires for this age group.

**Summary**

- This review describes 10 self‐report scales to assess youths’ Big Five traits.
- The 10 scales were developed or validated specifically for children or adolescents.
- The 10 scales differ for many characteristics (e.g., language, length, item type).
- We adapted the COSMIN guideline to assess validity and reliability aspects.
- The results can be useful for pursuing future research or developing new scales.

## 1 Introduction

Personality is defined as the dynamic organization of psychophysical systems determining the pattern of thoughts, feelings, and behaviors of each individual (Carver and Scheier 2012). The American Psychological Association (n.d.) describes it as “the enduring configuration of characteristics and behavior that comprises an individual's unique adjustment to life,” influenced by biological and social factors.

People's personality characteristics can be summarized in five factors, usually known as the “Big Five” traits (Goldberg 1993; McCrae and John 1992). A variety of studies assessed these dimensions from childhood to adulthood. However, while there are many self‐report scales for measuring them in adults, only a few self‐report questionnaires were developed specifically for children and/or adolescents. This systematic review aimed to describe the characteristics and some validity and reliability issues of questionnaires designed to assess self‐reported Big Five traits in young people.

### 1.1 The Big Five Model

In the scientific literature, personality has been conceptualized by a variety of approaches. Among them, one of the most utilized is the Big Five Model (BFM; Goldberg 1993)—alternative descriptions have been offered by the HEXACO (Ashton and Lee 2007) or temperament models (e.g., Rothbart and Bates 2006). This model proposes that personality can be organized into five major traits, that is, the so‐called Big Five. As reported in Goldberg's (1993) historical reconstruction, the BFM derived from two traditions. The first concerns the “lexical hypothesis,” which enabled to determine basic individuals' characteristics by analyzing the lexicon of many different languages (Allport and Odbert 1936; Galton 1884; Norman 1963). The second originated from a series of factorial analyses on standardized questionnaires about personality, which led to the identification of three main dimensions, then integrated by two other factors (McCrae and Costa 1985). The existence of these two traditions is among the reasons underlying the use of different labels for the same factor (John et al. 2008; McCrae and John 1992). Following one of the most widespread nomenclatures, Factor I is Extraversion, Factor II is Agreeableness, Factor III is Conscientiousness, Factor IV is Neuroticism, and Factor V is Openness. These five traits were described as “big” (Goldberg 1981) because they represent broad first‐order dimensions that can be distinguished into a variety of more specific second‐order facets (John et al. 2008): for example, sociability, assertiveness, activity, and positive emotionality for Extraversion; modesty, trust, tender‐mindedness, and altruism for Agreeableness; order, industriousness, reliability, and self‐discipline for Conscientiousness; anxiety, irritability, depression, and vulnerability for Neuroticism; and intellect, aesthetics, imagination/creativity, and adventurousness for Openness (Costa and McCrae 1992; Saucier and Ostendorf 1999; Soto and John 2009).

### 1.2 Big Five Traits in Children, Adolescents, and Adults

The BFM has been proven to be a valid model for adult personality studies (Caspi et al. 2005). However, an increasing amount of research applies the same model to describe children and adolescents' personality traits (Soto and Tackett 2015), and research evidence indicated differences and similarities between childhood, adolescence, and adulthood personality.

The first issue regards the comparability of personality characteristics of the same individual as a child and, later in life, as an adult. The Big Five traits are considered both changeable and stable over time, as they are influenced by genetics and environment (Shiner 2010). On the one hand, in line with the maturity principle (for which, during development, the average level of each trait tends to vary; Soto and Tackett 2015), Roberts et al.'s (2006) meta‐analytic results revealed that, at increasing ages, people become more socially dominant (an aspect related to Extraversion), more agreeable, more conscientious, and less neurotic. Similarly, a recent meta‐analysis of longitudinal studies found high rates of mean‐level change in Agreeableness, Conscientiousness, and Neuroticism (Bleidorn et al. 2022). However, this trend does not seem to apply to the Openness factor, as people show a decline in this trait after young adulthood (Caspi et al. 2005). On the other hand, consistently with the cumulative‐continuity principle (for which individuals maintain their position in the rank from the highest to the lowest for each trait; Soto and Tackett 2015), a quantitative review suggested increasing stability of personality traits over time (Roberts and Del Vecchio 2000). Similar results were found in Bleidorn et al.'s (2022) meta‐analysis. Coherently, a 50‐year longitudinal study involving more than 1700 Americans found mean‐level changes in the traits due to maturity processes but, at the same time, an almost stable personality profile from adolescence to old adulthood (Damian et al. 2019). Atherton et al. (2022), involving longitudinally for 12 years about 1100 Mexican adults, reported a certain rank order and profile stability and mean‐level declines in all the traits. However, it is yet unclear if such principles can completely describe what happens in the transition from childhood to adolescence. According to the disruption hypothesis (Denissen et al. 2013; Soto and Tackett 2015), in those periods, the maturity process in personality could be characterized by temporary dips due to biological, social, and psychological changes. More research with children and adolescents is needed to better explore the processes at the basis of personality development (Laceulle et al. 2023).

A second issue concerns differences between young people's and adults' personality structures. On the one hand, some evidence suggests that the five factors are not always detectable in infants and young children. Research about temperament (a construct often used to describe individual differences during childhood; Rothbart and Bates 2006) revealed the existence of three major traits, that is, Surgency, Effortful Control, and Negative Affectivity (somehow similar to Extraversion, Conscientiousness, and Neuroticism; De Pauw et al. 2009; Morizot 2014; Rothbart and Bates 2006; Shiner 2010). Instead, other authors proposed a six‐factor model for describing youths' personality traits, calling them the “Little Six,” that is, Extraversion, Agreeableness, Conscientiousness, Neuroticism, Openness to Experience, and Activity (Soto 2016; Soto and John 2014). On the other hand, many studies converged in finding a similar five‐trait personality structure in children (at least, from the school age) and adults (De Pauw et al. 2009; Shiner 2010). This emerged when children's personality was assessed both through scales distinguishing five dimensions and with measures capturing a larger number of traits (Shiner 2010). However, the Big Five factors seem to have a diverse content in children compared to adults because their behaviors and experiences are different. This could explain, for example, the low reliability and validity of the Openness trait when assessed with children and adolescents (Caspi et al. 2005; Lamb et al. 2002; Mervielde et al. 1995; Mervielde and De Fruyt 2000). We also note that some authors prefer to use different labels for young people's personality factors, like Benevolence and Imagination instead of Agreeableness and Openness (Mervielde and De Fruyt 1999).

### 1.3 Measuring Big Five Traits in Children and Adolescents

Measuring adults' Big Five traits with valid and reliable instruments is possible, and many self‐report scales are available for this purpose. Examples are the *Big Five Inventory‐2* (BFI‐2; Soto and John 2017), the *Big Five Questionnaire* (BFQ; Caprara et al. 1993), and the *Revised NEO Personality Inventory* (NEO‐PI‐R; Costa and McCrae 1992). There are also many studies that investigated the Big Five personality dimensions in children and adolescents: For example, our initial search returned more than 500 records about this topic (see the Results section).

From a developmental perspective, it is important to consider the peculiarities of children and adolescents when assessing their personality (as well as other psychological constructs) for many reasons. First, it is fundamental to take into account their attention capacities. Research evidence indicates that attention span increases with age (Simon et al. 2023). As such, it is preferable to use short and less time‐consuming questionnaires with children, whereas the use of longer instruments is feasible with adolescents. Second, it is pivotal to focus on participants' levels of text comprehension and reading skills (Kaplan 2013). Younger children are facilitated when items are easy to understand, consist of short statements, and include a lexicon appropriate to their age. However, despite having an increased text comprehension ability, adolescents can also benefit from questionnaires using brief sentences and simple words. Moreover, the use of picture‐based instruments could be a further facilitating factor for young participants (Mpundu‐Kaambwa et al. 2024). Finally, specifically regarding personality assessment, questionnaires should describe situations that are familiar to the target samples (Barbaranelli et al. 2003).

#### 1.3.1 Approaches to Assess Children and Adolescents' Big Five Traits

Youths' Big Five traits are usually assessed following three approaches, that is, using self‐report scales originally developed for adults, using other‐report scales, and using self‐report scales specifically designed for children and adolescents (Maćkiewicz and Cieciuch 2016; Ortet et al. 2012).

The first approach consists of the use of instruments developed for adults to collect self‐report data from children and/or adolescents without any adaptation (e.g., De Fruyt et al. 2000; Jovanović 2019; Serrano et al. 2021). This method is useful for longitudinal designs in which the participants respond over a broad time interval or for cross‐sectional studies comparing youths and adults (De Fruyt et al. 2000), but it has some limitations. For example, young participants could experience difficulties in understanding some descriptors (De Fruyt et al. 2000; McCrae et al. 2005) and the items could reflect situations and aspects that are frequent or typical for adults but not for children or adolescents (Maćkiewicz and Cieciuch 2016; Vollrath et al. 2016).

The second approach is related to the use of other‐report measures. In the studies following this approach, parents or teachers were often involved as relevant informants about young people's personal characteristics, both with vantage and disadvantage points (Shiner et al. 2021). Parents have many occasions to observe their offspring, but often do not have a comparison group. On the contrary, teachers have access to a wide comparison group but have the possibility to observe the target only in a limited context and time. An example of an instrument used is the *Hierarchical Personality Inventory for Children* (HiPIC; Mervielde and De Fruyt 1999; Vollrath et al. 2016), a questionnaire specifically developed to measure children's Big Five traits through others' reports. This approach also permits gathering data with toddlers and pre‐school children who have not yet developed the ability to read and answer a questionnaire by themselves (Zupančič et al. 2003). Although this approach has some strengths, such as the reduction of socially desirable answers detectable in self‐report data, informants do not have access to the entire wealth of thoughts, feelings, and behaviors of another person, so their judgment could be inaccurate (Dodorico McDonald 2008).

The third approach regards the use of self‐report measures specifically developed for young people (e.g., Lounsbury et al. 2003) or scales developed for adults but adapted for children and adolescents (e.g., McCrae et al. 2005). Notwithstanding limitations such as the possible occurrence of social desirability bias, this approach can overcome the main disadvantages of the other two measurement types. These instruments take into account the cognitive developmental level and the verbal skills of the ages for which they are designed; their contents reflect personality characteristics and behaviors specific to childhood and adolescence, and they permit children and adolescents to judge their own personality characteristics, not always detectable from the outside. In addition, a meta‐analytic study comparing self‐ and other reports about Big Five personality traits revealed that judges were strongly similar for most of the factors, in contrast with the hypothesized self‐enhancement effect (Kim et al. 2019). However, it should be noted that self‐report measures are particularly challenging for very young children due to their reduced reading skills, their difficulties in comprehension, and their possible lack of the capacities to gain insight and to compare with others (Shiner et al. 2021).

Acknowledging that all three approaches have strengths and weaknesses, we decided to focus this systematic review on instruments belonging solely to the third one for many reasons. First, self‐report questionnaires are the most used method for measuring personality traits (Dodorico McDonald 2008). Second, from an applied perspective, it is a very economical method (Pekrun 2020) as it enables researchers to involve young participants in a simple and quick way (e.g., through data collection in school settings), also for longitudinal and large‐scale studies. Third, young people are often easier to recruit than parents or teachers (Black et al. 2021), so in some cases, other‐reports cannot be used. Fourth, specifically when using self‐reports, age‐appropriate instruments are important to facilitate item comprehension and to take into account the cognitive and experiential differences of people in various developmental stages.

### 1.4 Validity and Reliability of Children and Adolescents' Self‐Reports

An open issue regarding the measurement of the Big Five in children and adolescents concerns the evidence about the validity and reliability of their self‐reports.

Two types of validity and reliability that questionnaires should demonstrate are, respectively, structural validity and internal consistency. The former refers to the degree to which the scores of a questionnaire adequately reflect the dimensions of the examined construct, while the latter reflects the extent to which the items belonging to the same scale are intercorrelated (Mokkink et al. 2010). Soto et al. (2008) investigated whether children and adolescents could provide valid and reliable Big Five self‐reports with a large sample of 10–20‐year‐olds administering the BFI (John et al. 1991), a scale originally developed for adults that was also understandable for fifth graders (Benet‐Martínez and John 1998). After controlling for acquiescence—that is, the tendency to use a response style by agreeing or disagreeing with most items—they found the same five‐factor structure in all the considered age groups. Conversely, they documented age differences, with better results at age 20 compared to age 10, for within‐domain coherence—that is, the internal consistency given by high correlations between items of the same dimension—and for between‐domain differentiation—that is, the low correlations between items of different dimensions. In light of these results, it is important to consider the evidence about the structural validity and internal consistency of the self‐report questionnaires specifically designed for measuring Big Five traits in young people.

### 1.5 The Current Study

The aim of this study was to conduct a systematic review of self‐report questionnaires measuring Big Five personality traits in children and/or adolescents. First, we identified research articles describing the development or validation (i.e., testing the measurement properties and/or providing the translation in another language) of self‐report questionnaires specifically designed for assessing youths' Big Five. Second, we described their characteristics and the evidence supporting their structural validity and internal consistency.

## 2 Methods

### 2.1 Search Strategy and Inclusion/Exclusion Criteria

We conducted a systematic review considering three databases, that is, PsycINFO, PubMed, and Scopus, in November 2022. We used the following search string, combining keywords and Boolean operators: “(*big five* AND (child* OR adolescen*))”. Some keywords were truncated to search for various suffixes (e.g., searching adolescen*, we obtained the results for *adolescence*, *adolescent*, and *adolescents*). We limited the research using the following additional criteria: searching for (a) keywords only in the title and/or in the abstract; (b) journal articles published in peer‐reviewed journals; (c) studies involving under‐18‐year‐old participants; (d) English language journal articles. There were no restrictions on the time period.

We considered eligible those articles describing the development or the validation of self‐report questionnaires devoted to measuring children and/or adolescents' Big Five traits. We included articles about questionnaires: (a) developed for children and/or adolescents and (b) originally developed for adults and then adapted for children and/or adolescents (Ortet et al. 2012). We excluded articles that (a) were not focused on the Big Five traits; (b) used Big Five questionnaires with children and/or adolescents but did not develop or validate them; (c) validated preexisting adult Big Five questionnaires with at least a subsample of children and/or adolescents, without modifying the original questionnaire; (d) used Big Five questionnaires with adults; (e) described reviews or qualitative articles; (f) developed and/or validated other‐report questionnaires to measure children and/or adolescents' Big Five traits; and (g) described the development or the validation of Big Five instruments different from questionnaires.

After selecting the eligible articles from the three databases, we consulted their references and utilized the previously reported inclusion and exclusion criteria to identify other eligible articles.

### 2.2 Analysis of the Articles

**FIGURE 1:**
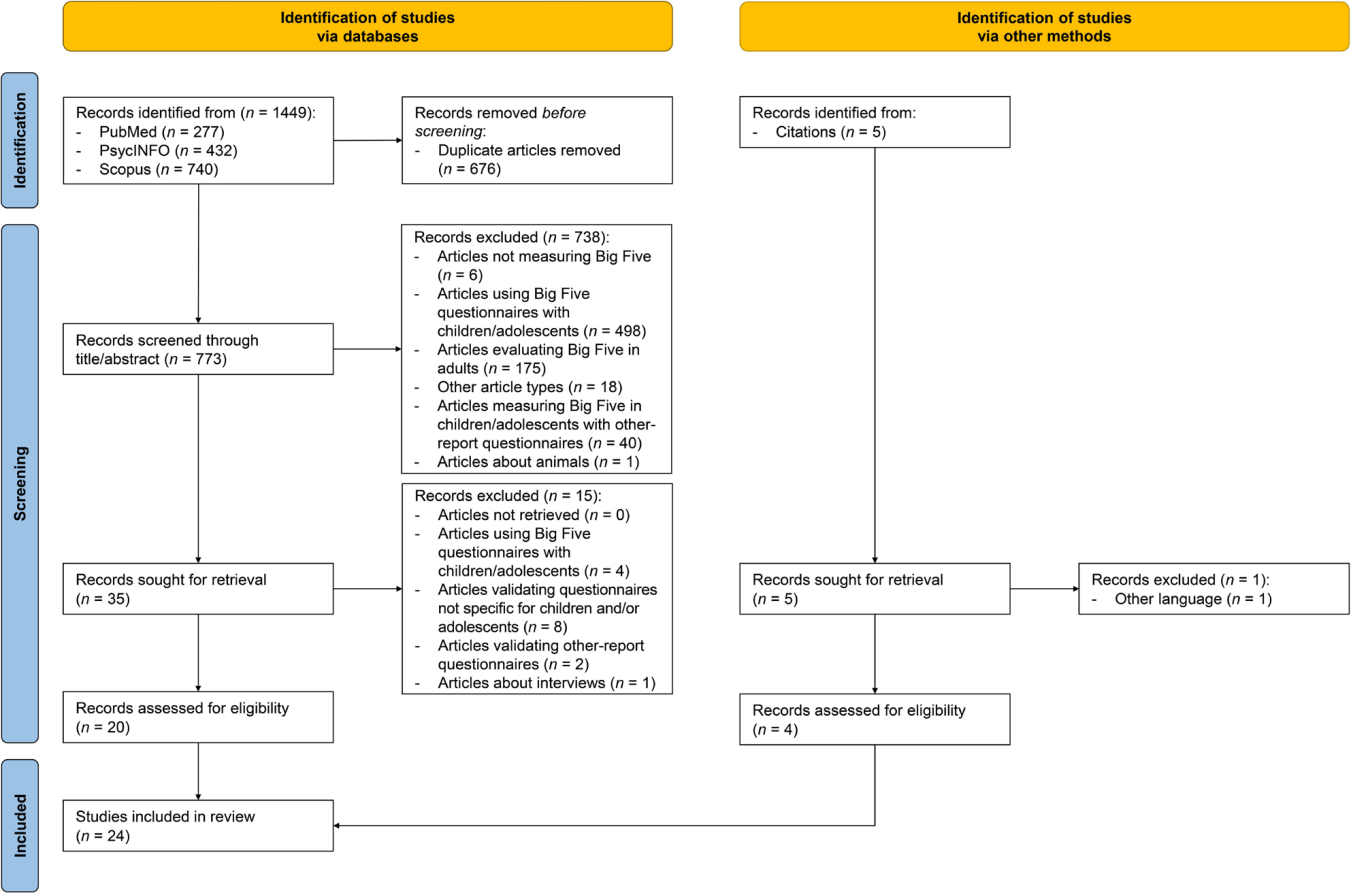
PRISMA diagram.

We analyzed the articles following the steps represented in the Preferred Reporting Items for Systematic reviews and Meta‐Analyses (PRISMA) diagram (Page et al. 2021; Figure 1). A first rater conducted the identification phase by downloading the references from the three databases. She found and removed duplicates using Microsoft Excel. The screening phase included different steps. The first author screened all the records based on titles and abstracts. A second rater screened 30% of them for reliability: The agreement was very high (Cohen's *k* = 0.89). Then, the first rater retrieved the full texts and selected those eligible to be included in the review. We also identified other eligible articles by consulting the references of the records considered eligible for the review. After identifying possibly relevant references, the first rater retrieved the full texts and selected other eligible records.

For each included article, we extracted data about the questionnaire's name, author/s, publication year, sample age or grades, language, dimensions, facets, total number of items, number of items per dimension, type of items, and response scale (Tables 1 and 2). When available, we extracted data about structural validity and internal consistency to assess the quality of the questionnaires (Table 3).

**TABLE 1:** Characteristics of the selected questionnaires.

Questionnaire	Author/s and year	Sample age or grades	Original language	Dimensions	Facets	Total number of items	Number of items for each dimension	Type of items	Response scale^a^
Big Five Questionnaire—Children version (BFQ‐C)	**Barbaranelli et al. (** 2003 **)**	Fourth‐eighth grades	Italian	Energy/Extraversion, Agreeableness, Conscientiousness, Emotional Instability, Intellect/Openness	No	65	13	Written statements	For elementary school: 3‐point scale For junior high school: 5‐point scale (1 = almost never, 5 = almost always)
	Barbaranelli et al. (2008)	13–14	Italian	—	—	—	—	—	5‐point scale
	Bore et al. (2020)	11–12	English	—	—	25^b^	5	—	3‐point scale
	Bouvard and Roulin (2017)	8–12	French	—	—	—	—	—	5‐point scale
	Cupani et al. (2020)	12–17	Spanish	—	—	—	—	—	5‐point scale
	del Barrio et al. (2006)	8–15	Spanish	—	—	—	—	—	5‐point scale
	Holgado‐Tello et al. (2009)	8–15	Spanish	—	—	—	—	—	5‐point scale
	Kokkinos and Markos (2017)	10–12	Greek	—	—	—	—	—	5‐point scale
	Kokkinos et al. (2020)	11–15	Greek	—	—	30	6	—	5‐point scale
	Markos and Kokkinos (2017)	11–16	Greek	—	—	30	6	—	5‐point scale
	Muris et al. (2005)	12–17	Dutch	—	—	—	—	—	5‐point scale
	Olivier and Herve (2015)	8–14	French	—	—	—	—	—	5‐point scale
	Robles‐Haydar et al. (2022)	11–16	Spanish	—	—	30	6	—	5‐point scale
Adolescent Personality Style Inventory (APSI)	**Lounsbury et al. (** 2003 **)**	11–18	English	Extraversion, Agreeableness, Conscientiousness, Neuroticism, Openness to Experience	No	55	11	Written statements	5‐point scale (1 = strongly disagree, 2 = disagree, 3 = in‐between, 4 = agree, 5 = strongly agree)
Pictorial Personality Traits Questionnaire for Children (PPTQ‐C)	**Maćkiewicz and Cieciuch (** 2016 **)**	7–13	Polish	Extraversion, Agreeableness, Conscientiousness, Neuroticism, Openness to Experience	No	15	3	Written bipolar statements and pictures	For 6–9‐year‐olds: 3‐point scale (2 = it depends) For 10–12‐year‐olds: 5‐point scale (1/5 = definitely yes, 2/4 = a little bit, 3 = it depends)
NEO Personality Inventory‐3 (NEO‐PI‐3)	**McCrae et al. (** 2005 **)**	14–20	English	Extraversion, Agreeableness, Conscientiousness, Neuroticism, Openness to Experience	Yes	240	48	Written statements	5‐point scale (1 = strongly disagree, 5 = strongly agree)
	Costa et al. (2008)	12–13	—	—	—	—	—	—	—
Big Five Personality Trait Short Questionnaire (BFPTSQ)	**Morizot (** 2014 **)**	12–15	French	Extraversion, Agreeableness, Conscientiousness, Emotional Stability, Openness	No	50	10	Written statements	5‐point scale (0 = totally disagree, 1 = disagree a little, 2 = neutral opinion, 3 = agree a little, 4 = totally agree)
	Ortet et al. (2022)	12–17	Spanish	—	—	—	—	—	—
Junior version of the Spanish NEO Personality Inventory–Revised (JS NEO)	**Ortet et al. (** 2012 **)**	12–18	Spanish	Extraversion, Agreeableness, Conscientiousness, Neuroticism, Openness to Experience	Yes	240	48	Written statements	5‐point scale (1 = strongly disagree, 5 = strongly agree)
SENNA1.0	**Primi et al. (** 2016 **)**	Fifth, 10th, and 12th grades	Brazilian	Extraversion, Agreeableness, Conscientiousness, Emotional Stability, Open‐Mindedness, Locus of Control/Negative Valence	No	For grades 10th–12th: 92 For grade fifth: 62	For the 92‐item version: 14 for Extraversion, 15 for Agreeableness, 17 for Conscientiousness, 14 for Emotional Stability, 17 for Open‐Mindedness, 15 for Locus of Control/Negative Valence	Written statements	5‐point scale
Five‐Factor Model Adolescent Personality Questionnaire (FFM‐APQ)	**Rogers and Glendon (** 2018 **)**	12–17	English	Extraversion, Agreeableness, Conscientiousness, Neuroticism, Openness	No	25	5	Written statements	5‐point scale (1 = strongly disagree, 5 = strongly agree)
THE WAY I AM	**Ruisel (** 1998 **)**	16–17	Slovak^c^	Introversion, Agreeability, Conscientiousness, Neuroticism, Openness to New Experiences	No	60	5	Adjectives	5‐point scale (1 = not true for me at all, 2 = true for me to a small extent, 3 = true for me to a medium extent, 4 = largely true for me, 5 = totally true for me)
—	**Scholte et al. (** 1997 **)**	12–18	Dutch	Extraversion, Agreeableness, Conscientiousness, Emotional Stability, Openness to Experience‐Intellect	No	25	5	Written bipolar statements	7‐point scale (1 = Pole A very true, 4 = both Pole A and Pole B a little bit true, 7 = Pole B very true)

*Note:* The references in bold indicate the articles about the development of the questionnaires; the other references indicate the validation studies. If not differently specified, the questionnaires used in the validation studies have the same characteristics of the original ones.

^a^ We reported the anchors specified within the article.

^b^ The items were reduced to 20 after factor analyses.

^c^ Information not directly specified within the original article.

**TABLE 2:** Summarized description of the selected questionnaires.

Questionnaire	Samples	Languages	Dimensions	Facets	Length^a^	Type of items	Response scale
Big Five Questionnaire—Children version (BFQ‐C)	Children and adolescents	Italian, English, French, Spanish, Greek, Dutch	Energy/Extraversion, Agreeableness, Conscientiousness, Emotional Instability, Intellect/Openness	Not measured	Long (65‐item version) and short (20/25 and 30‐item versions)	Written statements	3 or 5‐point scale
Adolescent Personality Style Inventory (APSI)	Adolescents	English	Extraversion, Agreeableness, Conscientiousness, Neuroticism, Openness to Experience	Not measured	Medium (55 items)	Written statements	5‐point scale
Pictorial Personality Traits Questionnaire for Children (PPTQ‐C)	Children and adolescents	Polish	Extraversion, Agreeableness, Conscientiousness, Neuroticism, Openness to Experience	Not measured	Short (15 items)	Written bipolar statements and pictures	3 or 5‐point scale
NEO Personality Inventory‐3 (NEO‐PI‐3)	Adolescents	English	Extraversion, Agreeableness, Conscientiousness, Neuroticism, Openness to Experience	Measured	Extra long (240 items)	Written statements	5‐point scale
Big Five Personality Trait Short Questionnaire (BFPTSQ)	Adolescents	French, Spanish	Extraversion, Agreeableness, Conscientiousness, Emotional Stability, Openness	Not measured	Medium (50 items)	Written statements	5‐point scale
Junior version of the Spanish NEO Personality Inventory–Revised (JS NEO)	Adolescents	Spanish	Extraversion, Agreeableness, Conscientiousness, Neuroticism, Openness to Experience	Measured	Extra long (240 items)	Written statements	5‐point scale
SENNA1.0	Children and adolescents	Brazilian	Extraversion, Agreeableness, Conscientiousness, Emotional Stability, Open‐Mindedness, Locus of Control/Negative Valence	Not measured	Long (62‐item version) and extra long (92‐item version)	Written statements	5‐point scale
Five‐Factor Model Adolescent Personality Questionnaire (FFM‐APQ)	Adolescents	English	Extraversion, Agreeableness, Conscientiousness, Neuroticism, Openness	Not measured	Short (25 items)	Written statements	5‐point scale
THE WAY I AM	Adolescents	Slovak	Introversion, Agreeability, Conscientiousness, Neuroticism, Openness to New Experiences	Not measured	Medium (60 items)	Adjectives	5‐point scale
Scholte et al.'s (1997) set	Adolescents	Dutch	Extraversion, Agreeableness, Conscientiousness, Emotional Stability, Openness to Experience‐Intellect	Not measured	Short (25 items)	Written bipolar statements	7‐point scale

^a^ We considered questionnaires with fewer than 30 items as *short*, between 31 and 60 items as *medium*, between 61 and 90 items as *long*, and more than 90 items as *extra long* (e.g., Sharma 2022).

**TABLE 3:** Assessment of each single analysis about structural validity and internal consistency of the selected questionnaires.

Questionnaire	Author/s and year	Language	Structural validity					Internal consistency			
			Statistical method	*n*	Methodological quality	Summarized results	(Rating) Index/indices	Statistical method	*n*	Methodological quality	(Rating) Index/indices
Big Five Questionnaire‐Children version (BFQ‐C)	**Barbaranelli et al. (** 2003 **)**	Italian	PCA	432 (elementary school children)	Adequate	Five factors; problems with some loadings	(?)				
			PCA	968 (junior high school children)	Adequate	Five factors; problems with some loadings	(?)				
			CFA (unrestricted factor analysis)	432 (elementary school children)	Doubtful	Five factors	(+) CFI = 0.87; RMSEA = 0.030				
			CFA (unrestricted factor analysis)	968 (junior high school children)	Doubtful	Five factors	(+) CFI = 0.90; RMSEA = 0.035				
	Barbaranelli et al. (2008)	Italian	CFA	386	Adequate	Five factors	(+) CFI = 0.99; RMSEA = 0.033; SRMR = 0.033	Alpha	386	Very good	(+) Range 0.82–0.95
	Bore et al. (2020)	English	EFA	13,707	Adequate	Five factors (25 items); problems with some loadings	(+) CFI = 0.98; TLI = 0.96; RMSEA = 0.03	Ordinal alpha (20‐item version)	13,708	Very good	EX (+): 0.70 AG (+): 0.74 CO (+): 0.78 NE (+): 0.81 OP (+): 0.86
			CFA	13,708	Very good	Five factors (20 items)	(+) CFI = 0.94; TLI = 0.93; RMSEA = 0.05				
	Bouvard and Roulin (2017)	French	CFA	399	Adequate	Five factors	(+) CFI = 0.82; RMSEA = 0.049				
			EFA	399	Adequate	Five factors; problems with some loadings	(+) CFI = 0.92; RMSEA = 0.03				

	Cupani et al. (2020)	Spanish	Rasch model (modifications to the original questionnaire)	1162	Very good	Five factors; some items deleted and some superitems created; reduced response scale for some dimensions	EX (−): Unidimensionality: per C < 5% = 1.38% Local independence: *r* < 0.20 Monotonicity: disordered thresholds Model fit: χ^2^ = 72.61	PSI (modified questionnaire)	1162	Very good	EX (−): 0.66 AG (+): 0.74 CO (+): 0.78 NE (+): 0.74 OP (+): 0.71
							AG (−): Unidimensionality: per C < 5% = 4.51% Local independence: *r* < 0.20 Monotonicity: disordered thresholds Model fit: χ^2^ = 43.30				
							CO (+): Unidimensionality: per C < 5% = 2.76% Local independence: *r* < 0.20 Monotonicity: ordered thresholds Model fit: *χ* ^2^ = 57.51				
NE (+): Unidimensionality: per C < 5% = 2.46% Local independence: *r* < 0.20 Monotonicity: ordered thresholds Model fit: *χ* ^2^ = 89.67											

						Five factors; some items deleted and some superitems created; reduced response scale for some dimensions
	OP (−): Unidimensionality: per C < 5% = 4.06% Local independence: *r* < 0.20 Monotonicity: disordered thresholds Model fit: χ^2^ = 49.48				
	del Barrio et al. (2006)					Spanish	CFA	Not specified (8–11‐year‐olds)	Adequate	Five factors	(+) CFI = 0.97; RMSEA = 0.072
							CFA	Not specified (12–15‐year‐olds)	Adequate	Five factors; modifications to the original questionnaire	(−) CFI = 0.90–0.92; RMSEA = 0.070–0.081
							CFA	487 (boys)	Very good	Five factors	(+) CFI = 0.96; RMSEA = 0.062
							CFA	337 (girls)	Adequate	Five factors	(−) CFI = 0.92; RMSEA = 0.08
	Holgado‐Tello et al. (2009)					Spanish	EFA	824	Adequate	Five factors; some items deleted; problems with some loadings	(?)
CFA							824	Very good	Four factors (56 items)	(−) CFI = 0.94; RMSEA = 0.069
Kokkinos and Markos 2017	Greek	CFA	1089	Very good	Bifactor model with five specific factors and a general factor	(+) CFI = 0.97; RMSEA = 0.034; SRMR = 0.052	Alpha	1089	Very good	EX (+): 0.75 AG (+): 0.81 CO (+): 0.81 NE (+): 0.82 OP (+): 0.76

	Kokkinos et al. (2020)	Greek	CFA	4747	Very good	Bifactor model with five specific factors and a general factor (30 items)	(+) CFI = 0.94; TLI = 0.93; RMSEA = 0.035	Omega (30‐item version)	4747	Very good	EX (+): 0.85 AG (+): 0.79 CO (+): 0.80 NE (+): 0.77 OP (+): 0.79
	Markos and Kokkinos (2017)	Greek	IRT	1089	Very good	Five factors; selected six items for each factor	(−) Unidimensionality: fit indexes not reported Local independence: *r* < 0.20 for each dimension Monotonicity: not investigated Model fit indexes: not reported	Omega	1089	Very good	EX (+): 0.79 AG (+): 0.83 CO (+): 0.83 NE (+): 0.83 OP (+): 0.80
			CFA	1089	Very good	Five factors; selected six items for each factor	(+) CFI = 0.95; TLI = 0.94; RMSEA = 0.039	Alpha	1089	Very good	EX (+): 0.75 AG (+): 0.81 CO (+): 0.81 NE (+): 0.82 OP (+): 0.76
			CFA	1089	Very good	Five factors (30 items)	(+) CFI = 0.98; TLI = 0.97; RMSEA = 0.042	Omega (30‐item version)	1089	Very good	EX (+): 0.80 AG (+): 0.83 CO (+): 0.83 NE (+): 0.83 OP (+): 0.80
CFA			347	Very good	Five factors (30 items)	(+) CFI = 0.98; TLI = 0.97; RMSEA = 0.042	Alpha (30‐item version)	1089	Very good	EX (+): 0.76 AG (+): 0.81 CO (+): 0.82 NE (+): 0.80 OP (+): 0.77

	
				
	Omega (30‐item version)	347						Very good	EX (+): 0.79 AG (+): 0.84 CO (+): 0.82 NE (+): 0.83 OP (+): 0.79
	Alpha (30‐item version)	347	Very good	EX (+): 0.73 AG (+): 0.70 CO (+): 0.79 NE (+): 0.76 OP (+): 0.76
	Muris et al. (2005)	Dutch	PCA	222	Inadequate	Five factors; problems with some loadings	(?)	Alpha	222	Very good	EX (+): 0.78 AG (+): 0.80 CO (+): 0.74 NE (+): 0.83 OP (+): 0.71
Olivier and Herve (2015)	French	PCA	386	Adequate	Five factors; problems with some loadings	(?)	Alpha	386	Very good	EX (+): 0.75 AG (+): 0.77 CO (+): 0.79 NE (+): 0.80 OP (+): 0.78
Robles‐Haydar et al. (2022)	Spanish	EFA	844	Adequate	Five factors (30 items)	(?)	Alpha (30‐item version)	844	Very good	EX (+): 0.74 AG (+): 0.71 CO (+): 0.73 NE (+): 0.85 OP (−): 0.64
		CFA	844	Very good	Five factors (30 items)	(+) CFI = 1.00; RMSEA = 0.053	Omega (30‐item version)	844	Very good	EX (+): 0.75 AG (+): 0.73 CO (+): 0.74 NE (+): 0.85 OP (−): 0.65
Adolescent Personality Style Inventory (APSI)	**Lounsbury et al. (** 2003 **)**	English	CFA	1339	Very good	Five factors (56 items)	(+) RMSEA = 0.059	Alpha	1353	Very good	EX (+): 0.81 AG (+): 0.79 CO (+): 0.75 NE (+): 0.81 OP (+): 0.73
			EFA	1339	Doubtful	Five factors (56 items)	(−) TLI = 0.82	Alpha	222	Very good	EX (+): 0.82 AG (+): 0.81 CO (+): 0.82 NE (+): 0.79 OP (+): 0.81
								Alpha	1061	Very good	EX (+): 0.85 AG (+): 0.82 CO (+): 0.84 NE (+): 0.85 OP (+): 0.80
Pictorial Personality Traits Questionnaire for Children (PPTQ‐C)	**Maćkiewicz and Cieciuch (** 2016 **)**	Polish	ESEM	501 (younger children)	Very good	Five factors; problems with some loadings	(+) CFI = 0.988; TLI = 0.969; RMSEA = 0.036	Alpha	501 (younger children)	Very good	EX (−): 0.60 AG (−): 0.69 CO (−): 0.65 NE (−): 0.69 OP (−): 0.48
			ESEM	527 (older children)	Very good	Five factors; problems with some loadings	(+) CFI = 0.990; TLI = 0.973; RMSEA = 0.035	Alpha	527 (older children)	Very good	EX (−): 0.50 AG (−): 0.67 CO (−): 0.61 NE (−): 0.62 OP (−): 0.44
NEO Personality Inventory‐3 (NEO‐PI‐3)	**McCrae et al. (** 2005 **)**	English	PCA	500	Inadequate	Five factors; problems with some loadings	(?)	Alpha	500	Very good	EX (+): 0.89 AG (+): 0.87 CO (+): 0.92 NE (+): 0.91 OP (+): 0.89^a^
	Costa et al. (2008)	English	PCA	202	Inadequate	Five factors; problems with some loadings	(?)	Alpha	202	Very good	EX (+): 0.88 AG (+): 0.86 CO (+): 0.93 NE (+): 0.90 OP (+): 0.84^b^
Big Five Personality Trait Short Questionnaire (BFPTSQ)	**Morizot (** 2014 **)**	French	ESEM (with correlated uniqueness)	1028	Very good	Five factors; problems with some loadings	(+) CFI = 0.932; TLI = 0.914; RMSEA = 0.029	Alpha	1028	Very good	EX (+): 0.80 AG (+): 0.72 CO (+): 0.80 NE (+): 0.81 OP (+): 0.71
	Ortet et al. (2022)	Spanish	ESEM (with correlated uniqueness)	1082	Very good	Five factors; problems with some loadings	(+) CFI = 0.934; TLI = 0.917; RMSEA = 0.031; SRMR = 0.030	Alpha	1082	Very good	EX (+): 0.80 AG (+): 0.74 CO (+): 0.78 NE (+): 0.80 OP (+): 0.77
								Omega	1082	Very good	EX (+): 0.80 AG (+): 0.73 CO (+): 0.80 NE (+): 0.81 OP (+): 0.74
Junior version of the Spanish NEO Personality Inventory–Revised (JS NEO)	**Ortet et al. (** 2012 **)**	Spanish	PCA	983	Inadequate	Five factors; problems with some loadings	(?)	Alpha	983	Very good	EX (+): 0.87 AG (+): 0.85 CO (+): 0.90 NE (+): 0.87 OP (+): 0.84^c^
SENNA1.0	**Primi et al. (** 2016 **)**	Brazilian	CFA	24,605	Very good	Six factors	(−) CFI = 0.700; TLI = 0.692; RMSEA = 0.064	Alpha	24,605	Very good	EX (+): 0.83 AG (+): 0.81 CO (+): 0.91 NE (+): 0.89 OP (+): 0.78
			ESEM	24,605	Very good	Six factors; problems with some loadings	(+) CFI = 0.915; TLI = 0.903; RMSEA = 0.036				
Five‐Factor Model Adolescent Personality Questionnaire (FFM‐APQ)	**Rogers and Glendon (** 2018 **)**	English	CFA	405	Very good	Five factors	EX (+): CFI = 0.994; TLI = 0.988; RMSEA = 0.036; SRMR = 0.021 AG (+): CFI = 1.000; TLI = 1.004; RMSEA = 0.000; SRMR = 0.014 CO (+): CFI = 0.978; TLI = 0.956; RMSEA = 0.061; SRMR = 0.032 NE (+): CFI = 0.977; TLI = 0.955; RMSEA = 0.072; SRMR = 0.030 OP (+): CFI = 0.965; TLI = 0.929; RMSEA = 0.067; SRMR = 0.033	Alpha	405	Very good	EX (+): 0.80 AG (+): 0.81 CO (+): 0.74 NE (+): 0.81 OP (+): 0.73
THE WAY I AM	**Ruisel (** 1998 **)**	Slovak	EFA	1051	Adequate	Five factors	(?)				
—	**Scholte et al. (** 1997 **)**	Dutch	PCA	2001	Adequate	Five factors; problems with some loadings	(?)	Alpha	2001	Very good	EX (+): 0.77 AG (+): 0.74 CO (−): 0.60 NE (+): 0.74 OP (−): 0.59
			CFA	2001	Doubtful	Five factors (oblique factors with secondary loading > |0.15|); problems with some loadings	(−) TLI = 0.90				

*Note:* The references in bold indicate the articles about the development of the questionnaires; the other references indicate the validation studies.

Abbreviations: +, sufficient; ?, doubtful; −, insufficient; AG, agreeableness; CFA, confirmatory factor analysis; CFI, comparative fit index; CO, conscientiousness; EFA, exploratory factor analysis; ESEM, exploratory structural equation modeling; EX, extraversion; NE, neuroticism; OP, openness; PCA, principal component analysis; Per C < 5%, proportion of *t*‐tests that were significant at the level of significance 0.05; PSI, person separation index; RMSEA, root mean square error of approximation; SRMR, standardized root mean square residuals; TLI, Tucker–Lewis index.

^a^ 9 out of 30 facets have an alpha value of < 0.70 (−).

^b^ 14 out of 30 facets have an alpha value of < 0.70 (−).

^c^ 25 out of 30 facets have an alpha value of < 0.70 (−).

### 2.3 Study Quality Assessment

We assessed the quality of the retrieved questionnaires by adapting a selection of the standards and criteria defined by the COSMIN (COnsensus‐based Standards for the selection of health Measurement INstruments) guideline for systematic reviews of Patient‐Reported Outcome Measures (PROMs; Mokkink et al. 2018; Prinsen et al. 2018; Terwee et al. 2018). The COSMIN standards help to evaluate the quality of each analysis on the basis of the designs and statistical methods (with five possible ratings: very good, adequate, doubtful, inadequate, and not applicable), and the COSMIN criteria help to assess the quality of the results (with three possible ratings: “+” if sufficient, “−” if insufficient, and “?” if indeterminate). We evaluated the validity and reliability of each study in terms of structural validity and internal consistency, respectively (Table 3; see the Supporting Information for a more detailed description of the quality assessment procedure and results; see Tables 1SI–3SI for the adapted COSMIN standards and criteria). Even though these guidelines were developed specifically for health‐related research and practice, they can also be adapted to other settings (e.g., Rezai et al. 2020).

## 3 Results

### 3.1 Study Selection

The initial search yielded 1449 research articles (Figure 1). We found 277 articles in PubMed, 432 in PsycINFO, and 740 in Scopus. We removed 676 duplicate records, and 773 articles were screened through their title and abstract. After this screening, a total number of 738 records were excluded: Six did not measure Big Five traits; 498 used adults' Big Five questionnaires with children and/or adolescents; 175 evaluated Big Five in adults; 18 were meta‐analyses, reviews, monographs, narrative reports, responses to other articles, or opening articles of special issues; 40 measured Big Five in children and/or adolescents through other‐report questionnaires; and 1 was focused on animals. As a result, 35 records were sought for retrieval. Among them, we excluded 15 records: Four only used Big Five questionnaires with children and/or adolescents; 8 validated questionnaires not specific for children and/or adolescents; 2 validated other‐report questionnaires; and 1 described an interview‐based instrument. At the end of this procedure, we considered 20 articles eligible.

After this first step, we consulted the references of these 20 articles for retrieving other eligible records. We identified 5 articles to be retrieved. One was excluded as it was not in English, and 4 were considered eligible.

In conclusion, we included in the systematic review a total number of 24 articles. Ten articles describe the development of 10 questionnaires (Table 1), that is, the *Big Five Questionnaire‐Children version* (BFQ‐C; Barbaranelli et al. 2003), the *Adolescent Personality Style Inventory* (APSI; Lounsbury et al. 2003), the *Pictorial Personality Traits Questionnaire for Children* (PPTQ‐C; Maćkiewicz and Cieciuch 2016), the *NEO Personality Inventory‐3* (NEO‐PI‐3; McCrae et al. 2005), the *Big Five Personality Trait Short Questionnaire* (BFPTSQ; Morizot 2014), the *Junior version of the Spanish NEO Personality Inventory–Revised* (JS NEO; Ortet et al. 2012), the *SENNA1.0* (Primi et al. 2016), the *Five‐Factor Model Adolescent Personality Questionnaire* (FFM–APQ; Rogers and Glendon 2018), the *THE WAY I AM* (Ruisel 1998), and the set of bipolar items developed by Scholte et al. (1997). The other 14 articles describe the validation (i.e., the measurement of the psychometric properties and/or the translation in other languages) of the BFQ‐C (Barbaranelli et al. 2008; Bore et al. 2020; Bouvard and Roulin 2017; Cupani et al. 2020; del Barrio et al. 2006; Holgado‐Tello et al. 2009; Kokkinos and Markos 2017; Kokkinos et al. 2020; Markos and Kokkinos 2017; Muris et al. 2005; Olivier and Herve 2015; Robles‐Haydar et al. 2022), the NEO‐PI‐3 (Costa et al. 2008), and the BFPTSQ (Ortet et al. 2022).

### 3.2 Characteristics of the Included Questionnaires

In the following paragraphs, we compared the questionnaires on the basis of their main characteristics. We took into account the samples involved in the studies, languages, dimensions, facets, number and type of items, response scale, and procedure used to develop items. Table 1 reports an extended description of the peculiarities of each study. A concise synthesis of the characteristics of the 10 questionnaires is reported in Table 2.

#### 3.2.1 Samples

Participants ranged from 7 (Maćkiewicz and Cieciuch 2016) to 20‐year‐olds (McCrae et al. 2005). McCrae et al.'s (2005) study was the only one involving also adults: Excluding it, the other samples included only children and/or adolescents, with a maximum age of 18 years (Lounsbury et al. 2003; Ortet et al. 2012; Scholte et al. 1997).

Some questionnaires were originally tested with both children and adolescents (participants were considered adolescents from 11 years or sixth grade), that is, the BFQ‐C (Barbaranelli et al. 2003), the PPTQ‐C (Maćkiewicz and Cieciuch 2016), and the SENNA1.0 (Primi et al. 2016). However, some translations of the BFQ‐C were validated only with adolescents, that is, the Dutch (Muris et al. 2005), the short English (Bore et al. 2020), the short Greek (Kokkinos et al. 2020; Markos and Kokkinos 2017), and the short Spanish (Robles‐Haydar et al. 2022) versions.

Other questionnaires were developed and/or validated involving only adolescent samples, that is, the APSI (Lounsbury et al. 2003), the NEO‐PI‐3 (Costa et al. 2008; McCrae et al. 2005), the BFPTSQ (Morizot 2014; Ortet et al. 2022), the JS NEO (Ortet et al. 2012), the FFM‐APQ (Rogers and Glendon 2018), the THE WAY I AM (Ruisel 1998), and the Scholte et al.'s (1997) set.

#### 3.2.2 Languages

The 10 questionnaires were developed in different languages. The original version of the BFQ‐C (Barbaranelli et al. 2003, 2008) was in Italian. It was then validated in other languages, that is, English (Bore et al. 2020), French (Bouvard and Roulin 2017; Olivier and Herve 2015), Spanish (Cupani et al. 2020; del Barrio et al. 2006; Holgado‐Tello et al. 2009; Robles‐Haydar et al. 2022), Greek (Kokkinos and Markos 2017; Kokkinos et al. 2020; Markos and Kokkinos 2017), and Dutch (Muris et al. 2005). The BFPTSQ (Morizot 2014) was originally developed in French and then validated in Spanish (Ortet et al. 2022). The APSI (Lounsbury et al. 2003), the NEO‐PI‐3 (McCrae et al. 2005), and the FFM‐APQ (Rogers and Glendon 2018) were developed in English, whereas the PPTQ‐C (Maćkiewicz and Cieciuch 2016), the JS NEO (Ortet et al. 2012), the SENNA1.0 (Primi et al. 2016), and the Scholte et al.'s set (Scholte et al. 1997) are, respectively, in Polish, Spanish, Brazilian, and Dutch. As for the THE WAY I AM (Ruisel 1998), the study does not explicitly specify the questionnaire's language. However, considering the author's affiliation, we can reasonably hypothesize that it was developed in Slovak.

#### 3.2.3 Dimensions

Most of the questionnaires measure only the Big Five factors, except for the SENNA1.0 (Primi et al. 2016), which also evaluates a sixth dimension called *Locus of Control/Negative Valence*. This is related to the fact that the authors did not aim to develop a personality questionnaire; they wanted to assess social and emotional skills and, conducting factor analyses, they noticed that five out of six factors modeled on personality Big Five traits. However, even though all the questionnaires assess the same five personality factors, the authors used a variety of labels to refer to them.

Nearly all the questionnaires call Factor I *Extraversion* (Lounsbury et al. 2003; Maćkiewicz and Cieciuch 2016; McCrae et al. 2005; Morizot 2014; Ortet et al. 2012; Primi et al. 2016; Rogers and Glendon 2018; Scholte et al. 1997). Similarly, Barbaranelli et al. (2003) refer to it as *Energy/Extraversion*. The questionnaire developed by Ruisel (1998) uses the label *Introversion*, emphasizing the opposite pole of the same dimension.

Factor II is labeled *Agreeableness* in all the questionnaires (Barbaranelli et al. 2003; Lounsbury et al. 2003; Maćkiewicz and Cieciuch 2016; McCrae et al. 2005; Morizot 2014; Ortet et al. 2012; Primi et al. 2016; Rogers and Glendon 2018; Scholte et al. 1997) with only one exception: The THE WAY I AM (Ruisel 1998) refers to this factor using the term *Agreeability*.

As for Factor III, all the questionnaires agree to use the same label, that is, *Conscientiousness*.

For labeling Factor IV, 7 questionnaires focus on the negative characteristics of this dimension, utilizing the terms *Neuroticism* (Lounsbury et al. 2003; Maćkiewicz and Cieciuch 2016; McCrae et al. 2005; Ortet et al. 2012; Rogers and Glendon 2018; Ruisel 1998) or *Emotional Instability* (Barbaranelli et al. 2003), whereas the other 3 questionnaires call it *Emotional Stability*, stressing the positive pole of the factor (Morizot 2014; Primi et al. 2016; Scholte et al. 1997).

Finally, Factor V appears to be the most controversial. The APSI (Lounsbury et al. 2003), the PPTQ‐C (Maćkiewicz and Cieciuch 2016), the NEO‐PI‐3 (McCrae et al. 2005), and the JS NEO (Ortet et al. 2012) describe it as *Openness to Experience*. Similarly, the THE WAY I AM (Ruisel 1998) and the Scholte et al.'s (1997) set use, respectively, the label *Openness to New Experience* and *Openness to Experience–Intellect*. The SENNA1.0 (Primi et al. 2016) adopted the term *Open‐Mindedness*, the BFPTSQ (Morizot 2014) and the FFM‐APQ (Rogers and Glendon 2018) *Openness*, and the BFQ‐C (Barbaranelli et al. 2003) *Intellect/Openness*. This last choice—in line with Scholte et al. (1997)—was justified by the authors focusing on the characteristics of children and young adolescents: They argue that the intellect component of the factor is more appropriate for participants of these ages as they usually have less exposure to intellectual experiences and cultural or social opportunities than adults (Costa and McCrae 1992).

#### 3.2.4 Facets

Only 2 out of 10 questionnaires permit obtaining scores differentiated according to some facets of the Big Five factors, that is, the NEO‐PI‐3 (McCrae et al. 2005) and the JS NEO (Ortet et al. 2012). They evaluate 30 facets, six for each factor: for Extraversion, warmth, gregariousness, assertiveness, activity, excitement seeking, and positive emotions; for Agreeableness, trust, straightforwardness, altruism, compliance, modesty, and tender‐mindedness; for Conscientiousness, competence, order, dutifulness, achievement striving, self‐discipline, and deliberation; for Neuroticism, anxiety, angry hostility, depression, self‐consciousness, impulsiveness, and vulnerability; and for Openness to Experience, fantasy, aesthetics, feelings, actions, ideas, and values.

However, even though they do not provide facet‐related scores, some questionnaires were developed taking them into account when selecting the items (Barbaranelli et al. 2003; Morizot 2014; Scholte et al. 1997).

#### 3.2.5 Number of Items

The questionnaires have a different number of items, both in general and for each dimension.

The longest questionnaires are the NEO‐PI‐3 (McCrae et al. 2005) and the JS NEO (Ortet et al. 2012), with 240 items, 48 for each factor and 8 for each facet. In addition to these items, these two inventories include seven other repeated items used for preliminary control of random responses but excluded from calculating the total scores.

Another very long measure is the SENNA1.0 (Primi et al. 2016), which comprises 92 items in the version for 10th–12th graders. This questionnaire also includes 15 items for measuring the additional dimension of *Locus of Control/Negative Valence*, so the total number of items evaluating the Big Five is 77. It is also the only questionnaire, among those included in this review, in which items are not equally distributed among the Big Five (i.e., it has 14, 15, 17, 14, and 17 items, respectively, for Extraversion, Agreeableness, Conscientiousness, Emotional Stability, and Open‐Mindedness). Primi et al. (2016) also developed a reduced version for fifth graders, containing 62 items, but they did not explicitly state their number for each dimension.

The shortest questionnaires are the PPTQ‐C (Maćkiewicz and Cieciuch 2016)—with 15 items, three for each factor—the FFM‐APQ (Rogers and Glendon 2018), and the Scholte et al.'s (1997) set—both comprising 25 items, five for each factor.

The remaining questionnaires (Barbaranelli et al. 2003; Lounsbury et al. 2003; Morizot 2014; Ruisel 1998) have between 50 and 65 items, equally divided for measuring each Big Five trait. Some shorter versions of the BFQ‐C were validated in English (25 items, then reduced to 20; Bore et al. 2020), Greek (30 items; Kokkinos et al. 2020; Markos and Kokkinos 2017), and Spanish (30 items; Robles‐Haydar et al. 2022).

#### 3.2.6 Type of Items

Comparing the questionnaires, we found different types of items.

The THE WAY I AM (Ruisel 1998) is the only questionnaire, among those selected, that uses a list of adjectives, half with positive and half with negative valence (e.g., *timid*).

Items are presented as written statements in the BFQ‐C (e.g., *I like to joke*; Barbaranelli et al. 2003), the APSI (e.g., *I have a lot of energy when I am around other people*; Lounsbury et al. 2003), the NEO‐PI‐3 (e.g., *I act forcefully and energetically*; McCrae et al. 2005), the BFPTSQ (e.g., *I see myself as a person who… Is extraverted, sociable*; Morizot 2014), the JS NEO (e.g., *I can't sit still in class*; Ortet et al. 2012), the FFM‐APQ (e.g., *I see myself as someone who… Is very sociable*; Rogers and Glendon 2018), and the SENNA1.0 (e.g., *Is full of energy*; Primi et al. 2016).

The Scholte et al.'s (1997) set and the PPTQ‐C (Maćkiewicz and Cieciuch 2016) have a bipolar structure: The former includes written bipolar items (e.g., *shy, reserved/likes being with others*), whereas the latter combines written sentences containing bipolar adjectives with two pictures representing the same unisex character engaging in opposite behaviors (e.g., *I usually play… on my own/with others*). Maćkiewicz and Cieciuch (2016) justified their choice to develop a pictorial questionnaire considering that 7–11‐year‐old children have not entirely built the ability to reason at an abstract level yet, being more capable of thinking concretely (Piaget 1960).

#### 3.2.7 Response Scale

Focusing on the response scale, we found that the questionnaires use various formats, in some cases depending on the age group. Scholte et al. (1997) used a 7‐point scale. A 5‐point scale was used in the junior high school version of the BFQ‐C (Barbaranelli et al. 2003), in the APSI (Lounsbury et al. 2003), in the 10–12‐year version of the PPTQ‐C (Maćkiewicz and Cieciuch 2016), in the NEO‐PI‐3 (McCrae et al. 2005), in the BFPTSQ (Morizot 2014), in the JS NEO (Ortet et al. 2012), in the SENNA1.0 (Primi et al. 2016), in the FFM‐APQ (Rogers and Glendon 2018), and in the THE WAY I AM (Ruisel 1998). A 3‐point scale was adopted for facilitating younger children in the elementary school version of the BFQ‐C (Barbaranelli et al. 2003) and in the 6–9‐year version of the PPTQ‐C (Maćkiewicz and Cieciuch 2016). All the validation studies about the BFQ‐C used a 5‐point scale, with the only exception being the one by Bore et al. (2020), which utilized a 3‐point scale.

Moreover, most of these scales measured the level of agreement with each item (Lounsbury et al. 2003; Maćkiewicz and Cieciuch 2016; McCrae et al. 2005; Morizot 2014; Ortet et al. 2012; Rogers and Glendon 2018; Ruisel 1998; Scholte et al. 1997), and only one scale measured the frequency with which a characteristic occurs (Barbaranelli et al. 2003). For the SENNA1.0 (Primi et al. 2016), the information about the focus of the response scale was missing.

#### 3.2.8 Development of the Items

The items included in the 10 questionnaires were developed or chosen taking into account different criteria. Most of the authors selected the items considering already existing measures and/or conducting a review of the literature (Barbaranelli et al. 2003; Lounsbury et al. 2003; McCrae et al. 2005; Morizot 2014; Ortet et al. 2012; Primi et al. 2016; Ruisel 1998; Scholte et al. 1997). For the PPTQ‐C (Maćkiewicz and Cieciuch 2016), the authors used a deductive method, and for the FFM‐APQ (Rogers and Glendon 2018), they used both an inductive and a deductive approach. In many cases, the lexicon of the items was adapted to make them more understandable (Lounsbury et al. 2003; McCrae et al. 2005; Morizot 2014; Ortet et al. 2012; Rogers and Glendon 2018) and to better reflect cultural aspects or descriptions more adequate for children and/or adolescents (Barbaranelli et al. 2003; Lounsbury et al. 2003; McCrae et al. 2005; Ortet et al. 2012).

We briefly describe the procedure utilized to develop or choose the items for each questionnaire as follows.

##### 3.2.8.1 Big Five Questionnaire—Children Version

It is not a mere adaptation of the adult BFQ (Caprara et al. 1993). Based on a preliminary study, Barbaranelli et al. (2003) appositely selected the items reflecting the personality‐related behavioral characteristics that are more appropriate to describe children and adolescents rather than adults.

##### 3.2.8.2 Adolescent Personality Style Inventory

Lounsbury et al. (2003) developed an initial pool of 91 items based on other instruments and a literature review. They also tried to make the items easy to understand for adolescents by using statements describing simple single activities, preferences, or dispositions and by avoiding referring to certain jobs or activities typical of particular cultures. Teachers, psychologists, and students then revised the items. The authors also estimated their readability by calculating the Flesch–Kincaid grade level (equal to 3.2) and the Flesch reading ease score (equal to 88.9).

##### 3.2.8.3 Pictorial Personality Traits Questionnaire for Children

Maćkiewicz and Cieciuch (2016) deductively developed a pool of 25 items describing behaviors and asked a professional graphic designer to draw the corresponding images. Then, they involved two personality psychologists in a validation step. The authors decided to use a combination of textual statements and pictures, considering the level of cognitive abilities of young children. Finally, they further selected the items by conducting a pilot study.

##### 3.2.8.4 NEO Personality Inventory‐3

It constitutes an adaptation for adolescents of the adult NEO‐PI‐R (Costa and McCrae 1992). As a first step, McCrae et al. (2005) identified 48 potentially problematic items in the original measure. For each of them, they formulated two alternatives: an item with an easier formulation and an item with new but still relevant content. Finally, they selected the most appropriate items, taking into account the relation with their facet.

##### 3.2.8.5 Big Five Personality Trait Short Questionnaire

Morizot (2014) used the English items of the BFI (John et al. 1991, 2008) available on the International Personality Item Pool website as a starting point, considering their formulation as short statements easily understandable by adolescents. Experts translated them into French, simplifying the language and adding descriptors to make each statement clear for 12–13‐year‐olds. Other adjustments were made, deleting and/or replacing inappropriate items and adding new items to cover relevant facets for each dimension.

##### 3.2.8.6 Junior Version of the Spanish NEO Personality Inventory‐Revised

Ortet et al. (2012) started to adapt the adult NEO‐PI‐R (Costa and McCrae 1992) before the English adolescent version NEO‐PI‐3 (McCrae et al. 2005) was available. For developing the JS NEO, they identified the problematic items by administering the NEO‐PI‐R to four 12‐year‐olds and by considering problems that emerged in previous studies. They identified 132 items to be modified: Forty items were adapted, making them more adherent to the English version of the NEO‐PI‐R rather than the Spanish version; the other 92 items were adapted using a lexicon adequate to adolescents or were made more suitable to the specific cultural context.

##### 3.2.8.7 SENNA1.0

Primi et al. (2016) aimed to develop a questionnaire to measure socioemotional skills in young people rather than personality. Through a literature review, they identified eight instruments, that is, the Locus of Control Scale (Nowicki and Strickland 1973), the Rosenberg Self‐Esteem Scale (Rosenberg 1979), the Strengths and Difficulties Questionnaire (Goodman 1997), the BFI (John et al. 1991), the Self‐Efficacy Questionnaire for Children (Muris 2001), the BFQ‐C (Barbaranelli et al. 2003), the Core Self‐Evaluations (Judge et al. 2003), and the Grit Scale (Duckworth and Quinn 2009). They administered these questionnaires to a sample of children and adolescents, and, through factor analyses, they identified six factors and selected the best eight items for each of them.

##### 3.2.8.8 Five‐Factor Model Adolescent Personality Questionnaire

Rogers and Glendon (2018) used a four‐step procedure to develop and select the items. First, they conducted focus groups with adolescents, collecting personality‐based descriptions, which then were evaluated and reduced by a group of experts. Second, they further refined this pool through item analysis. Third, they tested a pilot 50‐item form through exploratory factor analysis (EFA). Fourth, they tested the remaining 30 items through a series of confirmatory factor analyses (CFA) to select the final 25 items.

##### 3.2.8.9 THE WAY I AM

Ruisel (1998) did not describe extensively the procedure used to develop the THE WAY I AM. However, the author states that it was derived from a list used in psychodiagnostic practice. Such a list, initially consisting of 200 adjectives, was narrowed through psychometric methods to originate this questionnaire.

##### 3.2.8.10 Scholte et al.'s Set

Scholte et al. (1997) followed two steps. First, they identified five relevant facets for each factor. Second, they developed a bipolar item for each facet based on the items included in three older measures, that is, a Dutch test for measuring five personality factors (Elshout and Akkerman 1975), the Dutch version of the items for measuring Big Five in the California Child Q‐set (van Lieshout and Haselager 1994), and the Dutch version of Goldberg's markers (Goldberg 1992).

### 3.3 Validity and Reliability

The structural validity of the 10 questionnaires was explored or confirmed using a variety of statistical methods, that is, CFA, exploratory structural equation modeling (ESEM), EFA, principal component analysis (PCA), item response theory (IRT), and Rasch modeling. The internal consistency of most of the selected questionnaires was tested with Cronbach's (1951) α, ordinal α, McDonald's (1999) ω, and/or person separation index (PSI). The assessment is reported in Table 3. In the Supporting Information, we described in detail the procedure and the results of the analyses conducted in line with an adaptation of the COSMIN standards and criteria (Mokkink et al. 2018; Prinsen et al. 2018; Terwee et al. 2018).

#### 3.3.1 Summarized Evidence About Validity and Reliability

As anticipated in the Methods section, for each analysis, we evaluated the quality of the designs and statistical methods (following the COSMIN standards; see the Supporting Information) and the quality of the results (following the COSMIN criteria; see the Supporting Information).

First, we focused on the structural validity. Considering the COSMIN standards, we found at least an analysis of very good quality that confirmed the hypothesized factorial structure for the BFQ‐C (Bore et al. 2020; Cupani et al. 2020; del Barrio et al. 2006; Markos and Kokkinos 2017; Robles‐Haydar et al. 2022), the APSI (Lounsbury et al. 2003), the PPTQ‐C (Maćkiewicz and Cieciuch 2016), the BFPTSQ (Morizot 2014; Ortet et al. 2022), the SENNA1.0 (Primi et al. 2016), and the FFM‐APQ (Rogers and Glendon 2018). As regards the COSMIN criteria, the results of these analyses were considered sufficient in most cases. For the THE WAY I AM (Ruisel 1998) and the Scholte et al.'s (1997) set, the five factors emerged only in one analysis of adequate quality (COSMIN standards) but with indeterminate results (COSMIN criteria). Finally, the factorial structure of the NEO‐PI‐3 (Costa et al. 2008; McCrae et al. 2005) and the JS NEO (Ortet et al. 2012) was tested with analyses of inadequate quality (due to the small sample size, according to the COSMIN standards) whose results were indeterminate (following the COSMIN criteria).

Second, we examined the internal consistency. Considering the COSMIN standards, we found that the analyses were of very good quality for all the questionnaires except the THE WAY I AM (Ruisel 1998). As for the COSMIN criteria, the results were sufficient in most cases. Insufficient findings emerged for all the factors of the PPTQ‐C (Maćkiewicz and Cieciuch 2016), for many facets of the NEO‐PI‐3 (Costa et al. 2008; McCrae et al. 2005) and the JS NEO (Ortet et al. 2012), for Energy/Extraversion and Intellect‐Openness, respectively, in the Spanish modified and shorter versions of the BFQ‐C (Cupani et al. 2020; Robles‐Haydar et al. 2022), and for Conscientiousness and Openness to Experience‐Intellect in the Scholte et al.'s (1997) set.

## 4 Discussion

In the last decades, the study of personality according to the BFM has been extended to children and adolescents (Soto and Tackett 2015). Notwithstanding the amount of research available on this topic, there are still many open questions regarding, for example, similarities and differences between the different ages’ traits and the biological and environmental processes underlying personality development. For this reason, it is of pivotal importance to have valid and reliable questionnaires for assessing the Big Five in youths.

Notwithstanding the advantages of some other‐report instruments that rely on parents or teachers as relevant informants (Shiner et al. 2021), giving children and adolescents the possibility to self‐evaluate their personalities is crucial as they have access to their whole repertoire of thoughts, feelings, and behaviors, not always detectable from the outside. Although many studies utilized questionnaires originally developed for adults to gather self‐report data with young people, this methodology presents a series of disadvantages related to reading comprehension difficulties and descriptions not fitting with this specific age (De Fruyt et al. 2000; Maćkiewicz and Cieciuch 2016; McCrae et al. 2005; Vollrath et al. 2016).

To our knowledge, this is the first systematic review examining self‐report questionnaires designed or adapted for assessing children and/or adolescents' Big Five personality traits. We selected 24 articles describing the development or the validation of 10 questionnaires.

First, we analyzed them by taking into account a variety of descriptive characteristics. Considering the samples involved, most questionnaires were designed specifically for adolescents (i.e., participants from 11 years old or sixth grade). Only three questionnaires were tested also with children (i.e., the BFQ‐C, the PPTQ‐C, and the SENNA1.0). The selected questionnaires are in nine different languages. The BFQ‐C (Barbaranelli et al. 2003) is the questionnaire for which we found the highest number of validated translations. The questionnaires also differ in the labels used for referring to the main Big Five traits, reflecting different traditions in this research field (John et al. 2008; McCrae and John 1992) and also the attempt to better take into account the specificity of these ages (e.g., making reference to the Intellect component of Openness; Barbaranelli et al. 2003; Costa and McCrae 1992; Scholte et al. 1997). As for the structure, the questionnaires range from 15 to 240 items, to be evaluated on a 3‐, 5‐, or 7‐point scale. On the one hand, the shortest questionnaires can be useful for collecting data with younger children—who are also facilitated by a reduced number of response alternatives—and in contexts with time restrictions (e.g., schools). On the other hand, the longest questionnaires—the NEO‐PI‐3 (McCrae et al. 2005) and the JS NEO (Ortet et al. 2012)—have the advantage of deepening the study of personality, permitting the obtainment of differentiated scores for 30 second‐order facets, six for each first‐order factor. Finally, the 10 questionnaires include different types of items developed or selected considering already existing instruments, conducting a literature review, or following inductive and/or deductive procedures. The items consist of written statements or adjectives, sometimes presented in a bipolar format. The PPTQ‐C (Maćkiewicz and Cieciuch 2016) can be considered the most original questionnaire as it combines bipolar written sentences with pictures to make it easier for young children to understand the meaning of the items.

Second, we evaluated the selected questionnaires' structural validity and internal consistency following the COSMIN guideline (Mokkink et al. 2018; Prinsen et al. 2018; Terwee et al. 2018). The psychometric properties of the 10 questionnaires were investigated in many studies for the BFQ‐C (Barbaranelli et al. 2003). However, for the other questionnaires, only one or two studies were available. For this reason, the summarized results presented in this systematic review should be considered preliminary: More research is needed to better investigate their validity and reliability.

Structural validity was tested for each questionnaire. The hypothesized factorial structure was revealed or confirmed in most cases, with few exceptions. This result is in line with the findings of Soto et al. (2008), confirming that also children and adolescents can provide responses about their personality characteristics, with a structure comparable to adults. However, many questionnaires highlighted some problems with the Openness factor (i.e., the BFQ‐C, the PPTQ‐C, the BFPTSQ, and the Scholte et al.'s set). Such result is in line with other evidence suggesting that this dimension is the most controversial in this age group (Caspi et al. 2005; Lamb et al. 2002; Mervielde et al. 1995; Mervielde and De Fruyt 2000). More research is needed to disentangle the appropriateness and the nature of this factor for children and adolescents.

Internal consistency was tested at least once for each questionnaire, with the only exception of the THE WAY I AM (Ruisel 1998). The less reliable questionnaire is the PPTQ‐C (Maćkiewicz and Cieciuch 2016), probably due to the low number of items for each dimension. Other relevant reliability problems were found for the NEO‐PI‐3 (Costa et al. 2008; McCrae et al. 2005) and the JS NEO (Ortet et al. 2012) at the facet level. The internal consistency of the other questionnaires was high, except for some values below the threshold for the Spanish modified and short versions of the BFQ‐C (Cupani et al. 2020; Robles‐Haydar et al. 2022) and the Scholte et al.'s (1997) set. Even though Soto et al. (2008) documented an increasing internal consistency with age, such findings suggest that reliable data about the Big Five can also be gathered in children and adolescents through self‐reports.

In conclusion, the findings emerging from this systematic review can guide researchers and practitioners in selecting the most adequate questionnaires, taking into account aspects such as participants' ages, time constraints, and the specificity level of the assessment.

On the one hand, when the targets are children, the most valid and reliable questionnaires are the BFQ‐C—with the advantage of being available in many different validated languages—and the SENNA1.0. However, given the length of these two instruments, when the time is limited, a good alternative could be the PPTQ‐C, which is shorter and characterized by a more child‐friendly format—despite having some internal consistency problems.

On the other hand, when involving adolescents, other valid and reliable questionnaires—besides the BFQ‐C and the SENNA1.0—are the APSI, the BFPTSQ, and the FFM‐APQ. Among them, the APSI is particularly useful when time is reduced, due to its shortness. The other instruments—that is, the PPTQ‐C, the NEO‐PI‐3, the JS NEO, the THE WAY I AM, and the Sholte et al.'s set—demonstrated doubtful structural validity and/or internal consistency and would benefit from further research. However, notwithstanding these limitations, questionnaires such as the NEO‐PI‐3 and the JS NEO, could be particularly useful for those researchers or practitioners interested in a deeper understanding of adolescents' personality, as they permit extracting information about the single facets of each factor.

### 4.1 Limitations

This systematic review has a series of limitations. First, the initial search strategy included only English peer‐reviewed research articles indexed in three of the most used databases. However, additional questionnaires could have been retrieved considering other types of resources (e.g., non‐peer‐reviewed articles, books, chapters, presentations at conferences, or dissertations), extending the languages, and/or consulting other databases. Second, by focusing solely on self‐report questionnaires specifically designed or adapted for children and/or adolescents, we excluded both other types of self‐report measures (e.g., interviews) and also instruments belonging to the other two approaches, that is, self‐report scales designed only for adults and other‐report scales. We did not include self‐report questionnaires originally developed for adults, considering the importance of relying on age‐appropriate instruments for taking into account young people's peculiarities (Kaplan 2013; Simon et al. 2023). However, we acknowledge that some adult‐based measures—such as the International Personality Item Pool (IPIP; Goldberg 1999)—contain simple and short item formulations that could be easily used with young people (Mlačić et al. 2007). Moreover, we decided to exclude other‐report questionnaires, considering the benefits and large use of self‐reports in psychological assessment (Dodorico McDonald 2008; Pekrun 2020). Nevertheless, we recognize the advantages of other‐report instruments in some contexts (e.g., for assessing Big Five in very young children). Third, we searched for questionnaires based on the BFM, excluding other personality (e.g., HEXACO; Ashton and Lee 2007) or temperament models (e.g., Rothbart and Bates 2006). Fourth, we did not separate questionnaires for assessing Big Five personality traits in children and in adolescents due to the paucity of the selected instruments. However, given the peculiarities of each developmental stage, it is fundamental to take into consideration age differences.

#### 4.1.1 Future Research

Future reviews could compensate for some of the limitations of this study. First, they could include instruments belonging to the two approaches excluded in our work—self‐report scales designed only for adults and other‐report scales—to broaden the list of useful Big Five questionnaires when assessing young people. Second, they could also investigate which self‐report questionnaires for children and/or adolescents were developed and/or validated following other personality frameworks.

In addition, the questionnaires described in this systematic review were developed or validated mostly with participants from Western, educated, industrialized, rich, and democratic (WEIRD) countries. However, previous research raised doubts about the possibility of confirming the Big Five structure of personality in developing countries characterized by a different socioeconomic background (Laajaj et al. 2019). For such a reason, it is paramount that future studies further explore the validity and reliability of instruments (also using advanced analyses, such as the Rasch model; Burro et al. 2025) devoted to measuring Big Five personality traits involving underrepresented cultural contexts, languages, and samples (including also young children).

Finally, an open issue in the study of personality in young people is the stability of the traits. Among the 24 articles retrieved in this review, only one (Ortet et al. 2012) tested it by re‐administering the questionnaire after a month and calculating test–retest reliability. Future research should take into account this aspect to shed light on the dynamics of personality development.

## 5 Conclusion

This systematic review described 10 self‐report questionnaires for measuring Big Five traits in children and adolescents. The questionnaires differ in many characteristics, that is, target age, language, facet‐level assessment, length, type of items, and response scale. In addition, for some questionnaires, there is stronger evidence of structural validity and internal consistency compared to others. The description provided in this review could help researchers or practitioners interested in assessing youths' personalities select the most appropriate questionnaire for each sample and/or context.

## Author Contributions

**Giada Vicentini:** conceptualization, data curation, formal analysis, investigation, methodology, software, validation, visualization, writing – original draft; **Daniela Raccanello:** conceptualization, methodology, supervision, validation, writing – review and editing; **Roberto Burro:** conceptualization, methodology, supervision, validation, writing – review and editing.

## Ethics Statement

The authors have nothing to report.

## Consent

The authors have nothing to report.

## Conflicts of Interest

The authors declare no conflicts of interest.

## Supporting information


Data S1.

## Data Availability

The authors confirm that the data supporting the findings of this study are available within the article and its Supporting Information.
