# Supplementary material for: Self‐Report Questionnaires to Measure Big Five Personality Traits in Children and Adolescents: A Systematic Review
Source: Scand J Psychol. 2025 Apr 1;66(5):627–53. doi: 10.1111/sjop.13110 (PMC12423744; doi:10.1111/sjop.13110)
Supplement: Supplementary file 1 — Data S1. [file SJOP-66-627-s001.docx]

Supporting Information

Self-report questionnaires to measure Big Five personality traits in children and adolescents: A systematic review

# Study quality assessment procedure

For assessing the quality of the included questionnaires, we used a selection of standards and criteria suggested by the COSMIN (COnsensus-based Standards for the selection of health Measurements) guideline for systematic reviews of Patient‐Reported Outcome Measures (PROM; Table 1SI and 2SI; Mokkink et al., 2018; Prinsen et al., 2018; Terwee et al., 2018).

On the one hand, the COSMIN standards help in assessing the quality of the utilized designs and statistical methods (with five possible ratings: very good, adequate, doubtful, inadequate, and not applicable). The rating given to the methodology quality of each analysis (Table 3) was established by selecting the lower rating assigned to the standards (Table 1SI and 2SI). On the other hand, the criteria provide indications for evaluating the findings about the measurement properties of the questionnaires (with three possible ratings: “+” if sufficient, “-” if insufficient, and “?” if indeterminate).

We focused on two domains, i.e., validity and reliability. We decided to evaluate, respectively, the measurement properties pertaining to structural validity (defined by the COSMIN guideline as an aspect of construct validity) and internal consistency. Structural validity is the degree to which the scores of a questionnaire adequately reflect the dimensions of the examined construct (Mokkink et al., 2010) and is usually investigated through confirmatory factor analysis (CFA), exploratory factor analysis (EFA), item response theory (IRT), and/or Rasch model. Internal consistency indicates the extent to which the items belonging to the same scale are intercorrelated (Mokkink et al., 2010) and is usually evaluated through Cronbach’s (1951) alpha (α), ordinal α (Zumbo et al., 2007), and/or McDonald’s (1999) omega (ω), following the classical theory test (CTT), and person separation index (PSI), following the IRT or Rasch approach.

Even though the COSMIN guideline also suggests grading the overall quality of the evidence for each questionnaire (helpful in providing recommendations), we decided not to do this part because of the small number of studies available for most of the selected questionnaires.

## Adaptation of the COSMIN standards and criteria

We adapted some standards and criteria suggested by the COSMIN guideline (Mokkink et al., 2018; Prinsen et al., 2018; Terwee et al., 2018). The adapted COSMIN standards used for evaluating structural validity and internal consistency are described, respectively, in Table 1SI and 2SI. The adapted criteria for interpreting the corresponding results are reported in Table 3SI.

For structural validity, the COSMIN standards consider CFA as a “very good” statistical method and EFA as an “adequate” statistical method (Table 1SI). As many studies performed also other types of analyses for investigating the factorial structure of the questionnaires, we decided to broaden these indications. We rated the use of exploratory structural equation modelling (ESEM) as “very good”, being a statistical method that incorporates the best aspects of CFA and EFA (Marsh, 2014). The use of principal component analysis (PCA) was considered comparable to an EFA – as both methods have the aim to find components in a set of data, notwithstanding their remarkable differences (Bryant & Yarnold, 1995) – and for this reason we evaluated it as “adequate”. When the articles compared more than one model (e.g., testing the goodness-of-fit of models comprising different number of factors) using the same sample, we took into account only the one considered the best by the author/s (Bore et al., 2020; Holgado-Tello et al., 2009; Kokkinos & Markos, 2017; Kokkinos et al., 2020; Morizot, 2014; Ortet et al., 2022; Robles-Haydar et al., 2022; Rogers & Glendon, 2018; Scholte et al., 1997).

As for the COSMIN criteria concerning structural validity, we extended them to evaluate what is sufficient for the IRT or Rasch approach. We added new criteria to confirm the unidimensionality of each factor based on PCA of standardized residual correlations (Chou & Wang, 2010; Linacre, 2023; Raiche, 2005) or paired *t*-tests between two subsets of items, one with positive residual loadings and one with negative residual loadings on the first principal component (the *t*-tests aimed at verifying that the estimates for each person were significantly different; for verifying unidimensionality, not more than 5% of *t*-tests should be outside the range ±1.96; Tennant & Pallant, 2006), modified the criteria to confirm the absence of local dependence based on Yen’s Q3 (e.g., González-de la Torre et al., 2023), and partially modified the criteria for adequate model fits (i.e., χ^2^ > 0.01 was applied not only to IRT but also to Rasch models). For internal consistency, we added the threshold to rate the ordinal α (Zumbo et al., 2007) and the PSI (Wright & Masters, 1982; Wright & Stone, 1999). In addition, we considered satisfied the prerequisite of “at least low evidence for sufficient structural validity” when at least one analysis of structural validity was conducted.

# Validity and reliability of the selected questionnaires

## Structural validity

The factor structure was tested for all the questionnaires using a variety of statistical methods (Table 3).

Following a CTT approach, 14 articles performed CFA (Barbaranelli et al., 2003, 2008; Bore et al., 2020; Bouvard & Roulin, 2017; del Barrio et al., 2006; Holgado-Tello et al., 2009; Kokkinos & Markos, 2017; Kokkinos et al., 2020; Lounsbury et al., 2003; Markos & Kokkinos, 2017; Primi et al., 2016; Robles-Haydar et al., 2022; Rogers & Glendon, 2018; Scholte et al., 1997), 4 ESEM (Maćkiewicz & Cieciuch, 2016; Morizot, 2014; Ortet et al., 2022; Primi et al., 2016), 6 EFA (Bore et al., 2020; Bouvard & Roulin, 2017; Holgado-Tello et al., 2009; Lounsbury et al., 2003; Robles-Haydar et al., 2022; Ruisel, 1998), and 7 PCA (Barbaranelli et al., 2003; Costa et al., 2008; McCrae et al., 2005; Muris et al., 2005; Olivier & Herve, 2015; Ortet et al., 2012; Scholte et al., 1997). Eight articles used more than one statistical method to investigate structural validity: PCA and CFA (Barbaranelli et al., 2003; Scholte et al., 1997), EFA and CFA (Bore et al., 2020; Bouvard & Roulin, 2017; Holgado-Tello et al., 2009; Lounsbury et al., 2003; Robles-Haydar et al., 2022), or CFA and ESEM (Primi et al., 2016). Ruisel (1998) did not specify the type of factor analysis used for evaluating the structural validity of the THE WAY I AM; however, from the use of varimax rotation we can infer that it was an EFA.

The structure of the brief Greek version of the BFQ-C was also tested using an IRT approach (Markos & Kokkinos, 2017) and its Spanish translation through the Rasch model (Cupani et al., 2020).

Considering the sample size, when the studies followed the CTT approach, the number of participants was evaluated as “very good” or “adequate” in most cases. The sample size was rated as “inadequate” for the Dutch validation of the BFQ-C (Muris et al., 2005), the NEO-PI-3 (Costa et al., 2008; McCrae et al., 2005), and the JS NEO (Ortet et al., 2012). Moreover, the sample size was “very good” for the IRT analysis (Markos & Kokkinos, 2017) and the Rasch model (Cupani et al., 2020) performed for the Spanish and Greek versions of the BFQ-C.

In addition, some minor methodological flaws (corresponding to a “doubtful” rating) were identified for the CFA performed in the original BFQ-C (Barbaranelli et al., 2003) and the Scholte et al.’s (1997) set, and for the EFA performed in the APSI (Lounsbury et al., 2003), as the articles did not specify the estimation (in CFA) or the rotation method (in EFA).

As regards the results, the fit indexes for the EFA were reported only by Bore et al. (2020), Bouvard and Roulin (2017), and Lounsbury et al. (2003). In the former two, they were sufficient (“+”) while, in the latter, the index reported was insufficient (“-”). In all the other EFA and PCA, they were not specified, so the evidence is considered indeterminate (“?”). The fit indexes of CFA and ESEM were sufficient (“+”) in most cases. They were insufficient (“-”) for the CFA conducted on the Spanish BFQ-C by del Barrio et al. (2006; only for the 12-to-15-year-old and female samples) and by Holgado-Tello et al. (2009), on the SENNA1.0 (Primi et al., 2016), and on the Scholte et al.’s (1997) set. As for the IRT approach, in Markos and Kokkinos (2017) the results were rated insufficient (“-”) on the whole, as the fit indexes for the EFA conducted to verify unidimensionality were not reported, monotonicity was not investigated, and the fit indexes of the models were not specified. In Cupani et al. (2020) the findings of the Rasch model were rated sufficient (“+”) only for Conscientiousness and Negative Emotionality as, for the other three factors, the monotonicity assumption was violated.

## Internal consistency

Cronbach’s (1951) α, ordinal α, McDonald’s (1999) ω, and/or PSI were calculated in at least one study for each questionnaire, except the THE WAY I AM (Ruisel, 1998; Table 3).

Even though the article describing the development of the BFQ-C did not report any index about internal consistency (Barbaranelli et al., 2003), this questionnaire demonstrated to be a reliable questionnaire in subsequent validation studies. The α and ω values of the five traits in the 65-item questionnaire were sufficient (“+”) and ranged, respectively, from 0.71 to 0.95 (Barbaranelli et al., 2008; Kokkinos & Markos, 2017; Markos & Kokkinos, 2017; Muris et al., 2005; Olivier & Herve, 2015) and from 0.79 to 0.83 (Markos & Kokkinos, 2017). Moreover, the English 20-item version had acceptable ordinal α (from 0.70 to 0.86; Bore et al., 2020) and the Greek 30-item version was reliable as well, with α values ranging from 0.70 to 0.82 (Markos & Kokkinos, 2017) and ω values ranging from 0.77 to 0.85 (Kokkinos et al., 2020; Markos & Kokkinos, 2017). The Spanish 30-item BFQ-C showed sufficient (“+”) reliability for all the factors except Intellect/Openness (α = 0.64; ω = 0.65; Robles-Haydar et al., 2022). In addition, the PSI calculated for a modified version of this questionnaire ranged from 0.66 to 0.78 (Cupani et al., 2020): The less reliable dimension was Energy/Extraversion, with a value insufficient (“-”), below the threshold.

A sufficient (“+”) internal consistency of the five main traits emerged also for the APSI (α = 0.73-0.85; Lounsbury et al., 2003), the NEO-PI-3 (α = 0.84-0.93; Costa et al., 2008; McCrae et al., 2005), the BFPTSQ (α = 0.71-0.81 and ω = 0.73-0.81; Morizot, 2014; Ortet et al., 2022), the JS NEO (α = 0.84-0.90; Ortet et al., 2012), the SENNA1.0 (α = 0.78-0.91; Primi et al., 2016), and the FFM-APQ (α = 0.73-0.81; Rogers & Glendon, 2018).

The Scholte et al.’s (1997) set satisfied the criteria for a sufficient (“+”) internal consistency for Extraversion, Agreeableness, and Emotional Stability (α = 0.74-0.77) but not for Conscientiousness (α = 0.60) and Openness to Experience-Intellect (α = 0.59).

The PPTQ-C (Maćkiewicz & Cieciuch, 2016) is the questionnaire with the lower evidence for internal consistency given that all the factors had insufficient (“-”) α values ranging from 0.44 to 0.69.

Few reliability problems also emerged for the facets assessed in the NEO-PI-3 and the JS NEO. McCrae et al. (2005), Costa et al. (2008), and Ortet et al. (2012) found, respectively, that nine, 14, and 25 out of 30 facets had α values < 0.70.

## Summarized evidence about validity and reliability for each instrument

### Big Five Questionnaire – Children Version

The factorial structure of the BFQ-C was investigated through a variety of statistical methods. Five factors emerged from EFA or PCA and/or were confirmed through CFA for the Italian, French, Spanish, Greek, and Dutch 65-item versions (Barbaranelli et al., 2003, 2008; Bouvard & Roulin, 2017; del Barrio et al., 2006; Markos & Kokkinos, 2017; Muris et al., 2005; Olivier & Harve, 2015), and for the English, Spanish, and Greek shorter versions (Bore et al., 2020; del Barrio et al., 2006; Holgado-Tello et al., 2009; Markos & Kokkinos, 2017; Robles-Haydar et al., 2022), notwithstanding the presence of some indexes below the thresholds in one study (del Barrio et al., 2006) and some problems with some loadings (mainly regarding the Intellect/Openness factor; Barbaranelli et al., 2003; Bouvard & Roulin, 2017; Holgado-Tello et al., 2009; Muris et al., 2005; Olivier & Harve, 2015). The five factors were confirmed also for the Greek 65-item version through IRT analysis (although insufficient data were reported; Markos & Kokkinos, 2017) and for a modified version of the Spanish translation using the Rasch model (with monotonicity problems for three out of five dimensions; Cupani et al., 2020). However, conducting CFA, Holgado-Tello et al. (2009), Kokkinos and Markos (2017), and Kokkinos et al. (2020) found different factorial structures. For the Spanish shorter version, a four-factor model (obtained by redistributing the Intellect/Openness items in other factors) was considered better than the hypothesized one, despite insufficient fit indexes; and, for both the long and the short Greek versions, a bifactor model with five specific factors and a general factor was selected.

Even though the article about the development of the BFQ-C does not report data about its internal consistency (Barbaranelli et al., 2003), nine out of 12 validation studies calculated α, ordinal α, ω, and/or PSI for each of the five dimensions (no data was available in Bouvard & Roulin, 2017; del Barrio et al., 2006; Holgado-Tello et al., 2009). These indexes were appropriate (i.e., ≥ 0.70) in most cases, both for the 65-item and the shorter versions. Problems with the Intellect/Openness factor were identified for the short Spanish BFQ-C (Robles-Haydar et al., 2022). In addition, Cupani et al. (2020) found a PSI below the threshold for the Energy/Extraversion dimension after modifying the questionnaire through Rasch modelling.

### Adolescent Personality Style Inventory

The five-factor structure of the APSI emerged from both a CFA and an EFA (even though they were conducted using 56 items instead of the final 55 items). Lounsbury et al. (2003) found sufficient internal consistency in testing the questionnaires with three different samples.

### Pictorial Personality Traits Questionnaire for Children

Maćkiewicz and Cieciuch (2016), conducting separate ESEM for younger and older children, confirmed the five dimensions of the PPTQ-C; however, they found some loading problems, especially in the Openness to Experience items. As for internal consistency, the PPTQ-C appeared to be the questionnaire with the lowest α (ranging from 0.44 to 0.69), with the worst values found for Openness to Experience. Considering that their questionnaire has only three items for dimension, the authors discussed these results, explaining that α depends on the number of items (with higher values at increasing numbers).

### NEO Personality Inventory-3

The structural validity of the NEO-PI-3 was explored only through PCA and using small samples considering the high number of items (McCrae et al., 2005; Costa et al., 2008). Five factors emerged from the analyses despite a few problems with loadings. Concerning internal consistency, both studies found high α values for the main five factors. However, McCrae et al. (2005) and Costa et al. (2008) reported α values below the threshold for nine and 14 out of 30 facets, respectively.

### Big Five Personality Trait Short Questionnaire

ESEM with correlated uniqueness were conducted for both the French and Spanish versions of the BFPTSQ (Morizot, 2014; Ortet et al., 2022). They mostly confirmed the five factors of the questionnaire, but they also highlighted problems with some loadings (mainly, the Spanish version presents some problems in the Openness factor; Ortet et al., 2022). The internal consistency was good for both versions.

### Junior Version of the Spanish NEO Personality Inventory-Revised

The five-factor structure of the JS NEO was clearly recognized through a PCA, and the facets had higher loadings on their intended factor, despite important secondary loadings (Ortet et al., 2012). The questionnaire had sufficient α for the five main traits, but the values were below the threshold for 25 out of 30 facets.

### SENNA1.0

Primi et al. (2016) confirmed the six factors of the SENNA1.0 with both a CFA (although the fit indexes were below the thresholds) and an ESEM (despite some cross-loadings). As for internal consistency, the α values were high for all the five traits.

### Five-Factor Model Adolescent Personality Questionnaire

The CFA conducted separately for the five dimensions of the FFM-APQ confirmed its structure (Rogers & Glendon, 2018). The internal consistency of the factors was good.

### THE WAY I AM

Five factors were found through EFA in the THE WAY I AM (Ruisel, 1998). For this questionnaire, no data about internal consistency was available.

### Scholte et al.’s Set

Scholte et al. (1997) found five dimensions using a PCA, despite some loading problems, and, after testing different CFA models, confirmed the five-factor structure of the questionnaire (however, the fit index reported was below the threshold).

As for internal consistency, the set developed by Scholte et al. (1997) presents adequate values for three out of five dimensions (i.e., Extraversion, Agreeableness, and Emotional Stability). A poor internal consistency emerged for Conscientiousness and Openness to Experience-Intellect.

# References

References marked with an asterisk indicate studies included in the systematic review.

*Barbaranelli, C., Caprara, G. V., Rabasca, A., & Pastorelli, C. (2003). A questionnaire for measuring the Big Five in late childhood. *Personality and Individual Differences, 34*(4), 645–664. https://doi.org/10.1016/S0191-8869(02)00051-X

*Barbaranelli, C., Fida, R., Paciello, M., Di Giunta, L., & Caprara, G. V. (2008). Assessing personality in early adolescence through self-report and other-ratings a multitrait-multimethod analysis of the BFQ-C. *Personality and Individual Differences, 44*(4), 876–886. https://doi.org/10.1016/j.paid.2007.10.014

*Bore, M., Laurens, K. R., Hobbs, M. J., Green, M. J., Tzoumakis, S., Harris, F., & Carr, V. J. (2020). Item response theory analysis of the Big Five Questionnaire for Children–Short Form (BFC-SF): A self-report measure of personality in children aged 11–12 years. *Journal of Personality Disorders, 34*(1), 40–63. https://doi.org/10.1521/pedi_2018_32_380

*Bouvard, M., & Roulin, J. L. (2017). Exploratory factor analysis of the French version of the Big Five Questionnaire for Children (BFQ-C). *Swiss Journal of Psychology, 26*(3), 125–130. https://doi.org/10.1024/1421-0185/a000196

Bryant, F. B., & Yarnold, P. R. (1995). Principal-components analysis and exploratory and confirmatory factor analysis. In L. G. Grimm & P. R. Yarnold (Eds.), *Reading and understanding multivariate statistics* (pp. 99–136). American Psychological Association.

Chou, Y.-T., & Wang, W.-C. (2010). Checking dimensionality in item response models with principal component analysis on standardized residuals. *Educational and Psychological Measurement, 70*(5), 717–731. https://doi.org/10.1177/0013164410379322

*Costa Jr, P. T., McCrae, R. R., & Martin, T. A. (2008). Incipient adult personality: The NEO‐PI‐3 in middle‐school‐aged children. *British Journal of Developmental Psychology, 26*(1), 71–89. https://doi.org/10.1348/026151007X196273

Cronbach, L. J. (1951). Coefficient alpha and the internal structure of tests. *Psychometrika, 16*, 297–324. https://doi.org/10.1007/BF02310555

*Cupani, M., Morán, V. E., Ghío, F. B., Azpilicueta, A. E., & Garrido, S. J. (2020). Psychometric evaluation of the Big Five Questionnaire for Children (BFQ-C): A Rasch model approach. *Journal of Child and Family Studies, 29*, 2472–2486. https://doi.org/10.1007/s10826-020-01752-y

*del Barrio, V., Carrasco, M. Á., & Holgado, F. P. (2006). Factor structure invariance in the Children’s Big Five Questionnaire. *European Journal of Psychological Assessment, 22*(3), 158–167. https://doi.org/10.1027/1015-5759.22.3.158

González-de la Torre, H., González-Artero, P. N., Muñoz de León-Ortega, D., Lancha-de la Cruz, M. R., & Verdú-Soriano, J. (2023). Cultural adaptation, validation and evaluation of the psychometric properties of an obstetric violence scale in the Spanish context. *Nursing Reports, 13*(4), 1368–1387. https://doi.org/10.3390/nursrep13040115

*Holgado-Tello, F. P., Carrasco-Ortiz, M. Á., del Barrio-Gándara, M. V., & Chacón-Moscoso, S. (2009). Factor analysis of the Big Five Questionnaire using polychoric correlations in children. *Quality & Quantity, 43*, 75–85. https://doi.org/10.1007/s11135-007-9085-3

*Kokkinos, C. M., & Markos, A. (2017). The Big Five Questionnaire for Children (BFQ-C). Factorial invariance across sex and age in a Greek sample of preadolescents. *European Journal of Psychological Assessment, 33*(2), 129–133. https://doi.org/10.1027/1015-5759/a000273

*Kokkinos, C. M., Markos, A., Michaelides, M. P., & Voulgaridou, I. (2020). Disentangling the factorial structure of the Greek Big Five Questionnaire for Children–Short Form. *Personality and Individual Differences, 156*, 109742. https://doi.org/10.1016/j.paid.2019.109742

Linacre, J. M. (2023). *A user’s guide to Winsteps Ministep. Rasch-model computer programs (version 5.6.4)*. https://www.winsteps.com/a/Winsteps-Manual.pdf

*Lounsbury, J. W., Tatum, H., Gibson, L. W., Park, S. H., Sundstrom, E. D., Hamrick, F. L., & Wilburn, D. (2003). The development of a Big Five adolescent personality inventory. *Journal of Psychoeducational Assessment, 21*(2), 111–133. https://doi.org/10.1177/073428290302100201

*Maćkiewicz, M., & Cieciuch, J. (2016). Pictorial Personality Traits Questionnaire for Children (PPTQ-C)—A new measure of children’s personality traits. *Frontiers in Psychology, 7*, 498. https://doi.org/10.3389/fpsyg.2016.00498

*Markos, A., & Kokkinos, C. M. (2017). Development of a short form of the Greek Big Five Questionnaire for Children (GBFQ-C-SF): Validation among preadolescents. *Personality and Individual Differences, 112*, 12–17. https://doi.org/10.1016/j.paid.2017.02.045

Marsh, H. W., Morin, A. J., Parker, P. D., & Kaur, G. (2014). Exploratory structural equation modeling: An integration of the best features of exploratory and confirmatory factor analysis. *Annual Review of Clinical Psychology, 10*, 85–110. https://doi.org/10.1146/annurev-clinpsy-032813-153700

*McCrae, R. R., Costa, Jr., P. T., & Martin, T. A. (2005). The NEO–PI–3: A more readable revised NEO personality inventory. *Journal of Personality Assessment, 84*(3), 261–270. https://doi.org/10.1207/s15327752jpa8403_05

McDonald, R. P. (1999). *Test theory: A unified treatment*. Erlbaum.

Mokkink, L. B., De Vet, H. C., Prinsen, C. A., Patrick, D. L., Alonso, J., Bouter, L. M., & Terwee, C. B. (2018). COSMIN risk of bias checklist for systematic reviews of patient-reported outcome measures. *Quality of Life Research, 27*, 1171–1179. https://doi.org/10.1007/s11136-017-1765-4

Mokkink, L. B., Terwee, C. B., Patrick, D. L., Alonso, J., Stratford, P. W., Knol, D. L., Bouter, L. M., & de Vet, H. C. (2010). The COSMIN study reached international consensus on taxonomy, terminology, and definitions of measurement properties for health-related patient-reported outcomes. *Journal of Clinical Epidemiology, 63*(7), 737–745. https://doi.org/10.1016/j.jclinepi.2010.02.006

*Morizot, J. (2014). Construct validity of adolescents’ self-reported Big Five personality traits: Importance of conceptual breadth and initial validation of a short measure. *Assessment, 21*(5), 580–606. https://doi.org/10.1177/1073191114524015

*Muris, P., Meesters, C., & Diederen, R. (2005). Psychometric properties of the Big Five Questionnaire for Children (BFQ-C) in a Dutch sample of young adolescents. *Personality and Individual Differences, 38*(8), 1757–1769. https://doi.org/10.1016/j.paid.2004.11.018

*Olivier, M., & Herve, M. (2015). The Big Five Questionnaire for Children (BFQ-C): A French validation on 8- to 14-year-old children. *Personality and Individual Differences, 87*, 55–58. https://doi.org/10.1016/j.paid.2015.07.030

*Ortet, G., Ibáñez, M. I., Moya, J., Villa, H., Viruela, A., & Mezquita, L. (2012). Assessing the five factors of personality in adolescents: The Junior version of the Spanish NEO-PI-R. *Assessment, 19*(1), 114–130. https://doi.org/10.1177/1073191111410166

*Ortet, G., Mezquita, L., Morizot, J., Ortet-Walker, J., & Ibáñez, M. I. (2022). Assessment of “los pequeños” Big Five: The Spanish version of the Big Five Personality Trait Short Questionnaire in adolescents. *Psychological Assessment, 34*(5), e32–e44. https://doi.org/10.1037/pas0001119

*Primi, R., Santos, D., John, O. P., & De Fruyt, F. (2016). Development of an inventory assessing social and emotional skills in Brazilian youth. *European Journal of Psychological Assessment, 32*(1), 5–16. https://doi.org/10.1027/1015-5759/a000343

Prinsen, C. A., Mokkink, L. B., Bouter, L. M., Alonso, J., Patrick, D. L., De Vet, H. C., & Terwee, C. B. (2018). COSMIN guideline for systematic reviews of patient-reported outcome measures. *Quality of Life Research, 27*, 1147–1157. https://doi.org/10.1007/s11136-018-1798-3

Raiche, G. (2005). Critical eigenvalue sizes in standardized residual principal components analysis. *Rasch Measurement Transactions, 19*(1), 1005–1012.

*Robles-Haydar, C. A., Amar-Amar, J., & Martínez-González, M. B. (2022). Validation of the Big Five Questionnaire (BFQ-C), short version, in Colombian adolescents. *Salud Mental, 45*(1), 29–34. https://doi.org/10.17711/SM.0185-3325.2022.005

*Rogers, M. E., & Glendon, A. I. (2018). Development and initial validation of the Five-Factor Model Adolescent Personality Questionnaire (FFM–APQ). *Journal of Personality Assessment, 100*(3), 292–304. https://doi.org/10.1080/00223891.2017.1303776

*Ruisel, I. (1998). Lexical hypothesis in a psychodiagnostic context. *Studia Psychologica, 40*(4), 244–249.

*Scholte, R. H., van Aken, M. A., & van Lieshout, C. F. (1997). Adolescent personality factors in self-ratings and peer nominations and their prediction of peer acceptance and peer rejection. *Journal of Personality Assessment, 69*(3), 534–554. https://doi.org/10.1207/s15327752jpa6903_8

Tennant, A., & Pallant, J. F. (2006). Unidimensionality matters! (A tale of two Smiths?). *Rasch Measurement Transactions, 20*(1), 1048–1051.

Terwee, C. B., Prinsen, C. A., Chiarotto, A., Westerman, M. J., Patrick, D. L., Alonso, J., Bouter, L. M., de Vet, H. C. W., & Mokkink, L. B. (2018). COSMIN methodology for evaluating the content validity of patient-reported outcome measures: A Delphi study. *Quality of Life Research, 27*, 1159–1170. https://doi.org/10.1007/s11136-018-1829-0

Wright, B. D., & Masters, G. N. (1982). *Rating scale analysis. Rasch measurement*. MESA press.

Wright, B. D., & Stone, M. (1999). *Measurement essentials (2nd ed.)*. Wide Range.

Zumbo, B. D., Gadermann, A. M., & Zeisser, C. (2007). Ordinal versions of coefficients alpha and theta for Likert rating scales. *Journal of Modern Applied Statistical Methods, 6*(1), 4. https://doi.org/10.22237/jmasm/1177992180

Table 1SI. Adaptation of the COSMIN standards for assessing structural validity (adapted from Mokkink et al., 2018; Prinsen et al., 2018; Terwee et al., 2018).

|  | **Very good** | **Adequate** | **Doubtful** | **Inadequate** | **Not applicable** |
| --- | --- | --- | --- | --- | --- |
| 1. For CTT: Was EFA or CFA or a similar analysis performed? | CFA OR ESEM performed | EFA OR PCA performed | - | No CFA or ESEM or EFA or PCA performed | Not applicable |
| 2. For IRT/Rasch: Does the chosen model fit the research question? | The chosen model fits well with the research question | Assumable that the chosen model fits well with the research question | Doubtful if the chosen model fits well with the research question | Chosen model does not fit the research question | Not applicable |
| 3. Was the sample size included in the analysis adequate? | FA: 7 times the number of items, and ≥ 100 | FA: at least 5 the times number of items, and ≥ 100; OR at least 6 times the number of items, but < 100 | FA: 5 times the number of items, but < 100 | FA: < 5 times the number of items | - |
|  | Rasch/1PL models: ≥ 200 subjects | Rasch/1PL models: 100-199 subjects | Rasch/1PL models: 50-99 subjects | Rasch/1PL models: < 50 subjects | - |
|  | 2PL (or more) parametric IRT models  or Mokken scale analysis: ≥ 1000 subjects | 2PL (or more) parametric IRT models  or Mokken scale analysis: 500-999 subjects | 2PL (or more) parametric IRT models or Mokken scale analysis: 250-499 subjects | 2PL (or more) parametric IRT models or Mokken scale analysis: < 250 subjects | - |
| 4. Were there any other important flaws in the design or statistical methods of the study? | No other important methodological flaws | - | Other minor methodological flaws (e.g., rotation/estimation method not described) | Other important methodological flaws (e.g., inappropriate rotation/estimation method) | - |

*Note.* CFA = confirmatory factor analysis; CTT = classical test theory; EFA = exploratory factor analysis; ESEM = exploratory structural equation modelling; FA = factor analysis; IRT = item response theory; PCA = principal component analysis; 1PL = one-parameter logistic; 2PL = two-parameter logistic.

Table 2SI. Adaptation of the COSMIN standards for assessing internal consistency (adapted from Mokkink et al., 2018; Prinsen et al., 2018; Terwee et al., 2018).

|  | **Very good** | **Adequate** | **Doubtful** | **Inadequate** | **Not applicable** |
| --- | --- | --- | --- | --- | --- |
| 1. Was an internal consistency statistic calculated for each unidimensional scale or subscale separately? | Internal consistency statistics calculated for each unidimensional scale or subscale | - | Unclear whether scale or subscale is unidimensional | Internal consistency statistic NOT calculated on a unidimensional scale | - |
| 2. For continuous scores: Was Cronbach’s α or ordinal α or McDonald’s ω calculated? | Cronbach’s α OR ordinal α OR McDonald’s ω calculated | - | Only item-total correlations calculated | No Cronbach’s α or ordinal α or McDonald’s ω and no item-total correlations calculated | Not applicable |
| 3. For dichotomous scores: Was Cronbach’s α or KR-20 calculated? | Cronbach’s α OR KR-20 calculated | - | Only item-total correlations calculated | No Cronbach’s α or KR-20 and no item-total correlations calculated | Not applicable |
| 4. For IRT-based scores: Was SE(θ) or reliability coefficient of estimated latent trait value (e.g., PSI) calculated? | SE(θ) OR reliability coefficient calculated | - | - | SE(θ) or reliability coefficient NOT calculated | Not applicable |

*Note.* IRT = item response theory; KR-20 = Kuder Richardson reliability; PSI = person separation index; SE(θ) = standard error of the theta; α = alpha; ω = omega.

Table 3SI. Adapted COSMIN criteria for evaluating the results related to structural validity and internal consistency (adapted from Mokkink et al., 2018; Prinsen et al., 2018; Terwee et al., 2018).

| **Measurement property** | **Rating** | **Criteria** |
| --- | --- | --- |
| Structural validity | + | *For CTT:*  CFA/ESEM/EFA: CFI or TLI or comparable measure > 0.95 OR RMSEA < 0.06 OR SRMR < 0.08  *For IRT/Rasch:*  No violation of unidimensionality: CFI or TLI or comparable measure > 0.95 OR RMSEA < 0.06 OR SRMR < 0.08 OR eigenvalue < 2 in PCA of standardized residual correlations OR *t*-tests outside the range ±1.96 < 5%  AND  No violation of local independence: residual correlations among the items after controlling for the dominant factor < 0.20 OR Q3 > 0.20 and < 0.30  AND  No violation of monotonicity: adequate looking graphs OR item scalability > 0.30  AND  Adequate model fit:  IRT/Rasch: χ^2^ > 0.01  OR  Rasch: infit and outfit mean squares ≥ 0.5 and ≤ 1.5 OR *Z*-standardized values > ‐2.00 and < 2.00 |
|  | ? | *For CTT:*  Not all information for “+” reported  *For IRT/Rasch:*  Model fit not reported |
|  | - | Criteria for “+” not met |
| Internal consistency | + | At least low evidence for sufficient structural validity  AND  Cronbach’s α or ordinal α or McDonald’s ω or PSI ≥ 0.70 for each unidimensional scale or subscale |
|  | ? | Criteria for “At least low evidence for sufficient structural validity” not met |
|  | - | At least low evidence for sufficient structural validity AND  Cronbach’s α or ordinal α or McDonald’s ω or PSI < 0.70 for each unidimensional scale or subscale |

*Note.* + = sufficient; ? = doubtful; - = insufficient; CFA = confirmatory factor analysis; CFI = comparative fit index; CTT = classical test theory; EFA = exploratory factor analysis; ESEM = exploratory structural equation modelling; IRT = item response theory; PCA = principal component analysis; PSI = person separation index; Q3 = Yen’s Q3; RMSEA = root mean square error of approximation; SRMR = standardized root mean square residuals; TLI = Tucker Lewis index; α = alpha; ω = omega.
